# Comparing the bacterial communities of wild and captive golden mantella frogs: Implications for amphibian conservation

**DOI:** 10.1371/journal.pone.0205652

**Published:** 2018-10-31

**Authors:** Luiza F. Passos, Gerardo Garcia, Robert J. Young

**Affiliations:** 1 School of Environment and Life Sciences, University of Salford Manchester, Salford, United Kingdom; 2 Chester Zoo, Cedar House, Upton by Chester, Chester, United Kingdom; University of Illinois at Urbana-Champaign, UNITED STATES

## Abstract

Bacterial communities are frequently found in symbiotic associations with most animal species. The characteristically moist amphibian skin provides a good environment for the growth of some species of bacteria; among these a few can act as a first line defense mechanism against infections. Amphibians in the wild have relatively high exposure to bacteria through environmental transmission and through interactions with different conspecifics, whilst in captivity animals interact with fewer individuals, as well as experiencing a less complex environment through which to obtain their bacterial community. Here we compared the skin microbiota of captive and wild *Mantella aurantiaca* to investigate whether the captive environment was affecting individuals’ skin associated bacteria. This could have survivorship implications if captive animals had a different skin microbial community in comparison to wild counterparts and they were to be used in a reintroduction program. The microbial community were characterized through 16S rRNA amplicon sequencing methodology. Analyses showed that captive individuals had significantly lower diversity of bacterial species and lower relative abundant microbiota when compared to wild populations; this could result in captive frogs released back to the wild probably has greater susceptibility to infections due to inadequate skin microbiota.

## Introduction

The global amphibian crisis has resulted in increased use of captive breeding as a conservation tool [1]. Maintaining captive populations is important in terms of species conservation for potential reintroduction into the wild [2]. However, there is evidence that the captive environment can have negative impacts on different aspects of amphibians’ ecology and behaviour, such as affecting their vocalizations [3], anti-predator responses [4] and skin microbiota [5], which could potentially affect the survival of released animals.

Bacterial communities are commonly found in symbiotic associations with most animal species [6,7]. Frequently, the bacterial community provides some sort of advantage to the host such as protection against pathogens [8], and in return, receives nutrients and a suitable microhabitat in which to live and reproduce [9]. The characteristically-moist amphibian skin surface provides a fertile environment for the growth of bacteria [7], some of which may be present throughout the life of the organism, and some of which that will vary according to environment drivers and life stage [10]. These symbiotic bacterial communities contribute to the innate immunity of the host amphibian via competitive interactions between species and the production of antimicrobial metabolites, which are able to control the growth of some potential pathogens [11]. Thus, they play an important role in protecting amphibians from infectious diseases, such as chytridiomycosis caused by the pathogenic *Batrachochytrium dendrobatidis* [11,12]. This pathogen has been found around the world as well as in different areas in Madagascar, the natural habitat of our study species [13].

The microbiota of amphibian skin is one of the defense mechanism this group has against infections [8,9,14, 15]. Therefore, the proper functioning of this symbiotic interaction between bacteria and amphibians is vital for captive individuals, which are due to be released back into the wild [14]. To understand whether captive bred frogs are fit for reintroduction, in terms of their skin microbiota, wild and captive frogs of the same species need to be compared. Antwis et al [14] observed changes in the richness and abundance of microbiota of captive *Agalychnis callidryas* when compared to their wild counterparts and, a similar result was also found in six species of Japanese amphibians [9] and for the Panamanian golden frog, *Atelopus zeteki* [15]. Kueneman et al. [5] has demonstrated the effect of captivity on the loss of skin-associated bacteria on frogs and increased chances of infections. The focus of this study is the golden mantella frog, a critically endangered and endemic species from Madagascar, which will have captive bred individuals reintroduced to boost wild populations’ numbers in a near future [16]. It is necessary to understand how captivity might have affect individuals to evaluate if animals are suitable candidates prior to release.

Amphibians in the wild have relatively high exposure to bacteria through environmental transmission and through interactions with both conspecifics and other species [17]. Amphibians in captivity interact with fewer individuals, as well as living in a less complex environment in which to obtain a rich and diverse bacterial community [14]. Husbandry guidelines for keeping amphibians include removing waste, cleaning substrate and using a bleach dilution on enclosures to avoid the risk of diseases, but this could lead to a more sterile environment [18]. Consequently, captive amphibians are likely to be exposed to a lower diversity of bacteria, and thus support a much simpler skin-associated bacterial community in comparison to their wild counterparts. This could potentially make them less resistant to diseases when being reintroduced to the wild environment [14,5].

During this research, we analysed how the unique set of conditions created by captive husbandry may affect golden mantella frogs’ (*Mantella aurantiaca)* skin microbial composition [1–3]. We predicted that captive bred frogs will have a different bacteria composition with a less rich skin microbiota than their wild counterparts.

## Methods

### Ethical approval

All the research reported in this study was approved by the Chester Zoo’s Ethics Committee, Ambatovy, University of Salford Science and Technology Ethics Panel (ST1617-82) and, it conforms to all regulations and laws in all relevant countries in relation to care of experimental animal subjects. Furthermore, we can confirm, from our post-experimental monitoring, that no animals suffered any injuries, became ill or were negatively affected as a result of this study.

### Study subjects

The model species for this study was the golden mantella frog (*Mantella aurantiaca)*. This is a species classified as critically endangered by the IUCN [19] and is endemic to the Moramanga district, in the Region of Alaotra-Mangoro, Madagascar. Its distribution is restricted to a fragment of forest surrounded by degraded land. A significant proportion of its population is located inside or near the area of the Ambatovy mine [20]. Gold mantella frogs are well known due to their aposematic orange-red colouration. Females are characteristically larger and heavier than males [20]. Following a conservation needs assessment, the Amphibian Ark prioritised *M*. *aurantiaca* as a species in need of *ex situ* assistance to safeguard its survival [21].

### Study sites

The data used for this study were obtained from captive (Chester Zoo, UK) and wild populations (two spatially independent wild populations of frogs). The captive colony has been in captivity for more than seven generations. Frogs are kept off show in a biosecurity container specifically for conservation-related research. Frogs are kept in a group of 16 individuals (10 males and 6 females), in a naturalistic tank with different live species of plants, moss for substrate, water, hiding places, UV light and heaters to mimic the natural conditions. Tanks are cleaned monthly using diluted total spectrum disinfectant (F10, Loughborough, UK). Wild frogs were sampled from Mangabe rainforest, a site of international biodiversity importance, home to most of the world’s breeding ponds for the golden mantella frog. The second wild population was from Ambatovy mining site, located within a species-rich region of Madagascar at the southern end of the remaining Eastern Forest Corridor in the Moramanga region. As part of the Environmental Management Plan, there is a Conservation Zone of native forest maintained by the mining company.

### Skin bacteria sampling

To analyse the bacterial composition on the skin of golden mantella frogs a standard protocol described by Antwis et al [14, 22] was followed. Sterile gloves were worn throughout handling and changed for each frog to minimize the risk of cross-contamination [14, 22]. Prior to specimen sampling, frogs were surface rinsed using sterile distilled water to remove any transient bacteria and ensure that the skin sampled included primarily skin-associated microbiota [14, 22]. Frogs were then swabbed for 20 seconds all over the entire body surface and limbs using sterile cotton-tipped collection swabs. Swabs were kept in Eppendorf tubes with 400 μl of QIAGEN ATL buffer (QIAGEN, UK) while in the field, another 200 μl of ATL buffer were added in the lab and samples were incubated at room temperature for two weeks prior to DNA extraction. Two weeks was the time between samples being collected in the wild and arriving to be processed in the laboratory, to avoid bias this incubation period was also added to samples collected in captivity. Care was taken to ensure frogs were not harmed during this process, individuals were kept in a plastic container after sampling to be monitored post-swabbing for signs of stress or injury in response to the swabbing (no adverse effects were observed) and to avoid re-sampling animals. After swabbing animals, we measured snout-vent length measured and body mass to allow for assessment of body condition a standard measure of amphibian health [23]. Wild populations were sampled on site to avoid the stress of translocating animals. All animals were released at the exact site they were collected.

### Molecular methods and sequencing analyses

During this study, we used culture-independent methodology for the characterization of the skin associated microbial community. A total of eight individuals from each population (4 males and 4 females), a total of 24 samples were used for the molecular analysis. All samples were collected during breeding season that occurs during the rainy period in Madagascar. DNA was extracted from the swabs using QIAGEN DNeasy Tissue and Blood kit (QIAGEN, UK). The standard QIAGEN protocol for swab samples was followed with modifications for samples with low quantities of DNA. Adjustments included 24 hour incubation at 56 °C after the addition of ATL buffer and Proteinase K. Addition of 4 μl of RNAse before adding AL Buffer and allowing AE buffer to sit on the filter for 20 min before the final elution [24]. A Thermo Scientific NanoDrop 2000 (Thermo Fisher Scientific, UK) spectrophotometer was used to determine the purity and DNA concentration of this pool.

Library preparation was done following the MiSeq 16S library preparation two step PCR Illumina protocol. Sampled bacteria community from captive and wild populations of *M*. *aurantiaca* were identified using the 16S Illumina amplicon protocol with primers 515F-806R (FWD:GTGCCAGCMGCCGCGGTAA; REV:GGACTACHVGGGTWTCTAAT) targeting the V4 [25]. 16S rRNA gene was amplified using a two stage PCR with a HotStart PCR kit (Kappa Biosystem, USA) following the manufacturer’s instructions. First stage PCR with the following program: 95°C for 3 min followed by 25 cycles of 95°C for 30 s, 55°C for 30 s, and 72°C for 30 s, had a final extension step of 5 minutes at 72°C. PCR products were checked for the correct length using a Tape Station Screen Tape High sensitivity (Agilent, USA) and then cleaned up using Agencourt AMPure XP beads (Beckman Coulter, UK), which were used to remove primer dimers.

A second stage PCR with the following program: 95°C for 3 min followed by 8 cycles of 95°C for 30 s, 55°C for 30 s, and 72°C for 30 s, with a final extension step of 5 minutes at 72°C, was used to attached Illumina adapters. PCR products were again checked for the correct length and then cleaned up to remove any unwanted DNA. Qubit Fluorometric Quantitation (Thermo Fisher Scientific, UK) was used to determine the purity and DNA concentration of each sample. Samples were pooled together and a qPCR using NEBNext Library Quantification Kit (Illumina, USA) was performed to quantify library DNA concentration. The library was loaded in the MiSeq Illumina using paired-end 2 x 250 V2 reagent cartridge with 10% PhiX (Illumina, USA) as control at the University of Salford, UK. A consensus sequence was obtained by combining the forward and reverse sequences and processed with the R package dada2 pipeline using the default parameters [26]. Consensus sequences were then blasted against the Ribosomal Database Project (RDP; http://rdp.cme.msu.edu/) to identify each bacterial OTU (Operational Taxonomic Unit) to genus level.

We used R packages Phyloseq and DESeq2 [27, 28] to import the OTU table to the R environment and to identify differences in the relative abundance of bacterial taxa between treatment groups using the DESeq2 nbinomWald function. This allows for detection of differential abundance patterns without the bias of rarefying libraries also avoiding omission of available valid data during analysis that would result in loss of sensitivity [28,29]. OTU relative abundance between the three populations and between wild and captive populations were quantified using Wald tests [28,29], a Bonferoni test was applied to correct p-values due to multiple testing. Alpha diversity was obtained using the Shannon-Wiener metric and compared between populations using an ANOVA test, and wild versus captive samples using a t-test. OTUs with <20 reads were removed from the data set and samples were rarefied to 9000 reads per samples.

Overall bacterial community composition was analysed for differences based on origin (wild versus captive) and population using the Adonis function of the vegan package [30] in RStudio [31]. Adonis is a permutational multivariate analysis that uses a Bray-Curtis distance matrix based on the abundance of each OTU to analyse the variation in the overall bacterial community structure. All tests were conducted in Rstudio version 0.99.903 (data for bacterial abundance were log transformed to achieve a normal distribution).

## Results

**Fig 1:**
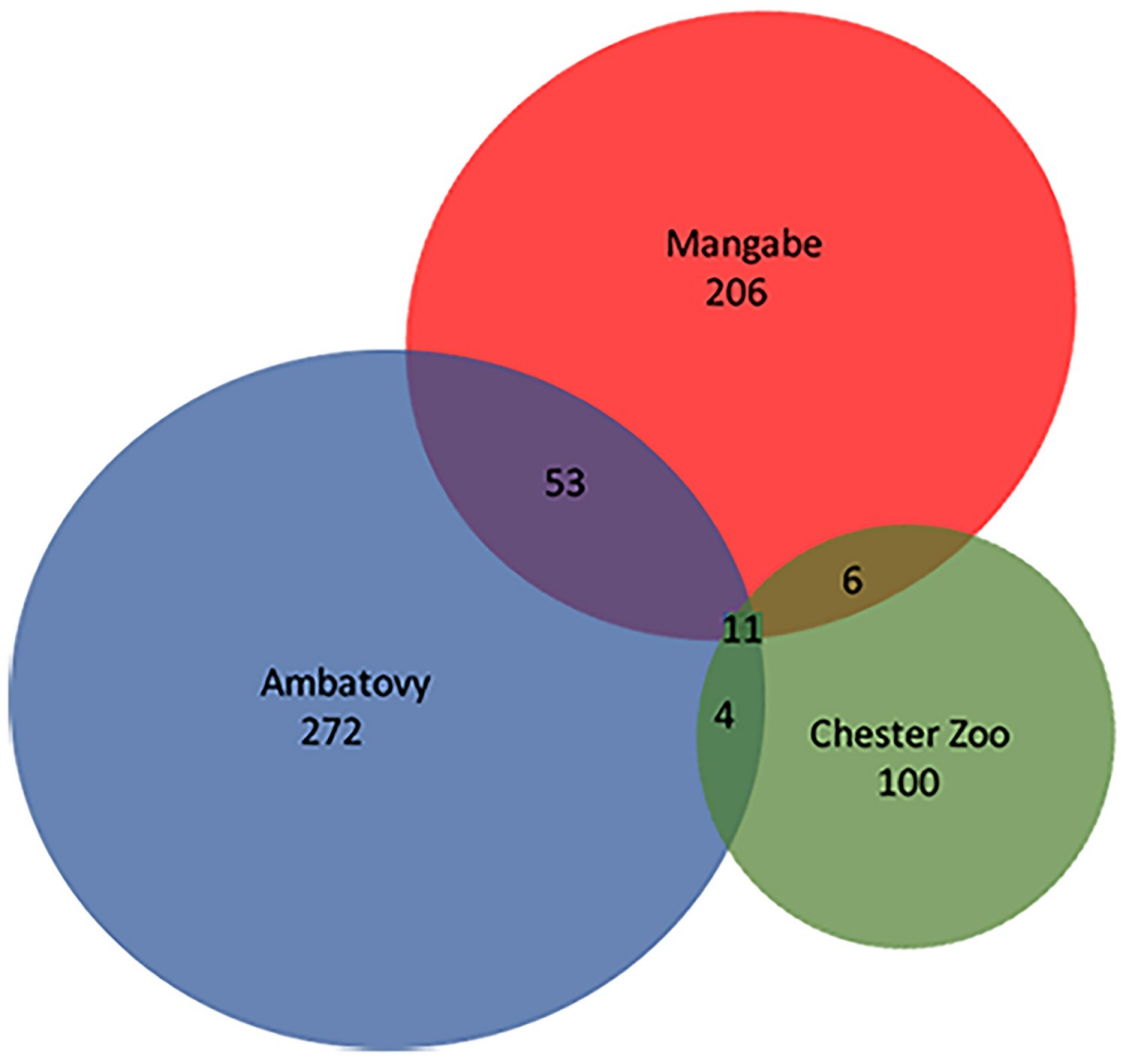
Graphical representation of shared and unique OTUs (Operational Taxonomic Units) of the three sampled populations of golden mantella frogs (Chester Zoo, Ambatovy and Mangabe), where the size of discs and overlaps among discs is proportional to the true number observed.

Analyses from the sequencing data showed 563 (S1 Appendix) different OTUs belonging to 20 phyla, 39 classes, 66 order, 98 families and 153 genera (Table 1). The mean number of sequences per sample was 14779±365 for Ambatovy samples, 17155± 419 for Mangabe samples and 9435±215 for samples from Chester Zoo. Two hundred and seventy-two OTUs were found from Ambatovy (wild) samples, 206 OTUs were found from the Mangabe (wild) population and only 100 OTUs from frogs kept at Chester Zoo (Fig 1). Some OTUs, across all populations, could not be identified due to poor a sequence.

**Table 1:** Number of phyla, classes, orders, families and genera of bacteria identified per golden mantella frog population.

Population	Origin	Phyla	Classes	Orders	Families	Genera
Ambatovy	Wild	11	21	38	65	87
Mangabe	Wild	20	39	60	84	114
Chester Zoo	Captive	9	15	23	34	40

**Fig 2:**
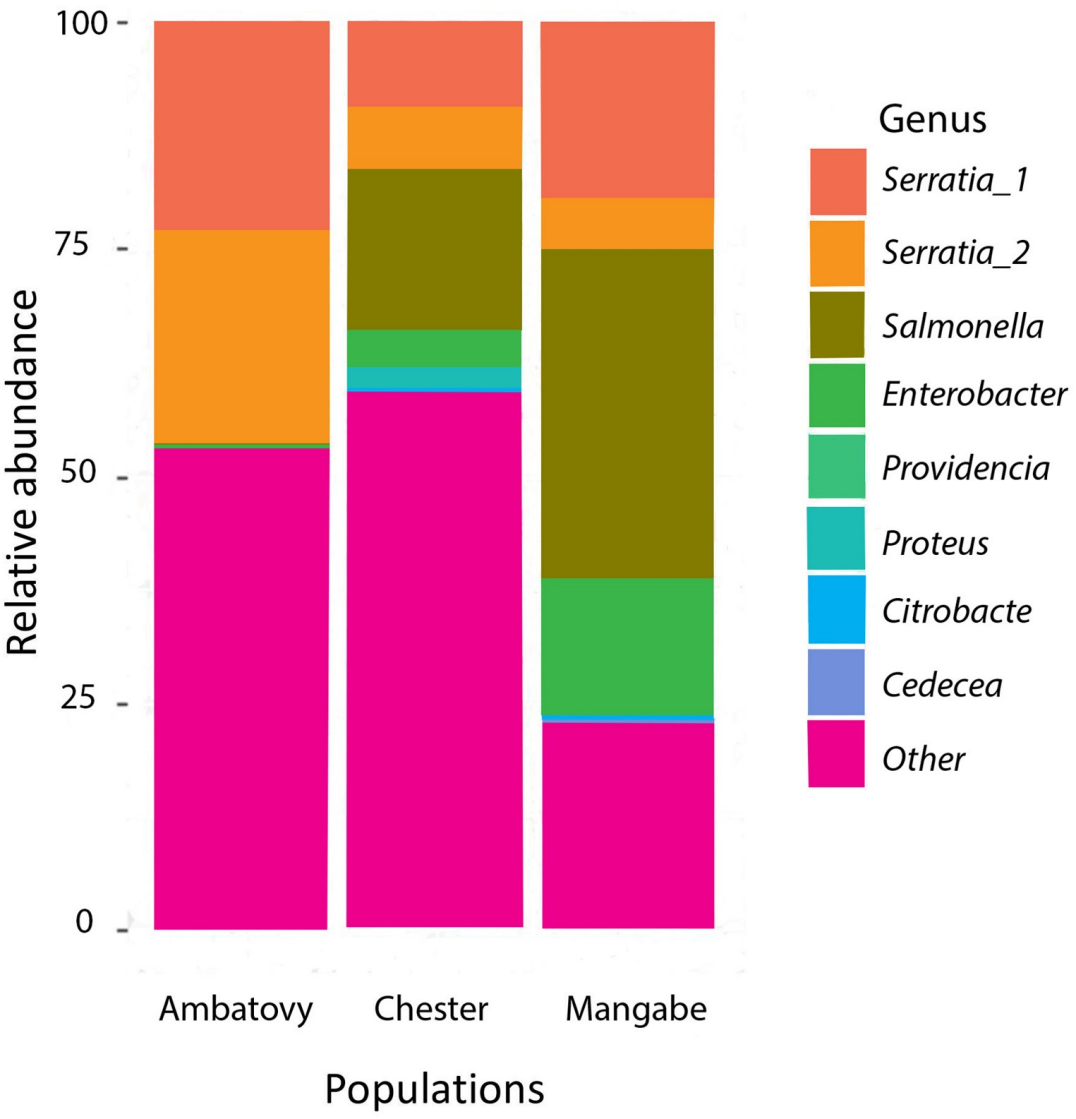
The relative abundance of sequences assigned to genera of major bacterial family (Enterobacteriacea) and all other genera observed in each of the golden mantella frog populations sampled (Chester Zoo, Ambatovy and Mangabe).

Only eleven bacterial genera (*Acinetobacter*, *Bradyrhizobium*, *Chryseobacterium*, *Dokdonella*, *Enterobacter*, *Providencia*, *Rubrobacter*, *Pseudomonas*, *Salmonella*, *Serratia* and *Spirosomo*) from six different families were found in the three populations. One family of bacteria, Enterobacteriaceae, comprised the greatest percentage of reads from both wild and captive *M*. *aurantiaca*, being the most abundant family (85% Mangabe, 76% Ambatovy and 60% Chester Zoo) (Fig 2).

Wild frogs had a significantly higher skin bacterial alpha diversity than those reared at Chester Zoo (wild Shannon-Wiener index = 56.55, captive Shannon-Wiener index = 11.83, t = 0.847, p<0.05). When alpha diversity was compared between the three populations using an ANOVA test, Mangabe (Shannon-Wiener index = 38.61) had the greatest diversity between all sampled populations (Ambatovy Shannon-Wiener index = 23.07, Chester Shannon-Wiener index = 11.83; F_1,22_ = 10.97, p<0.001).

**Fig 3:**
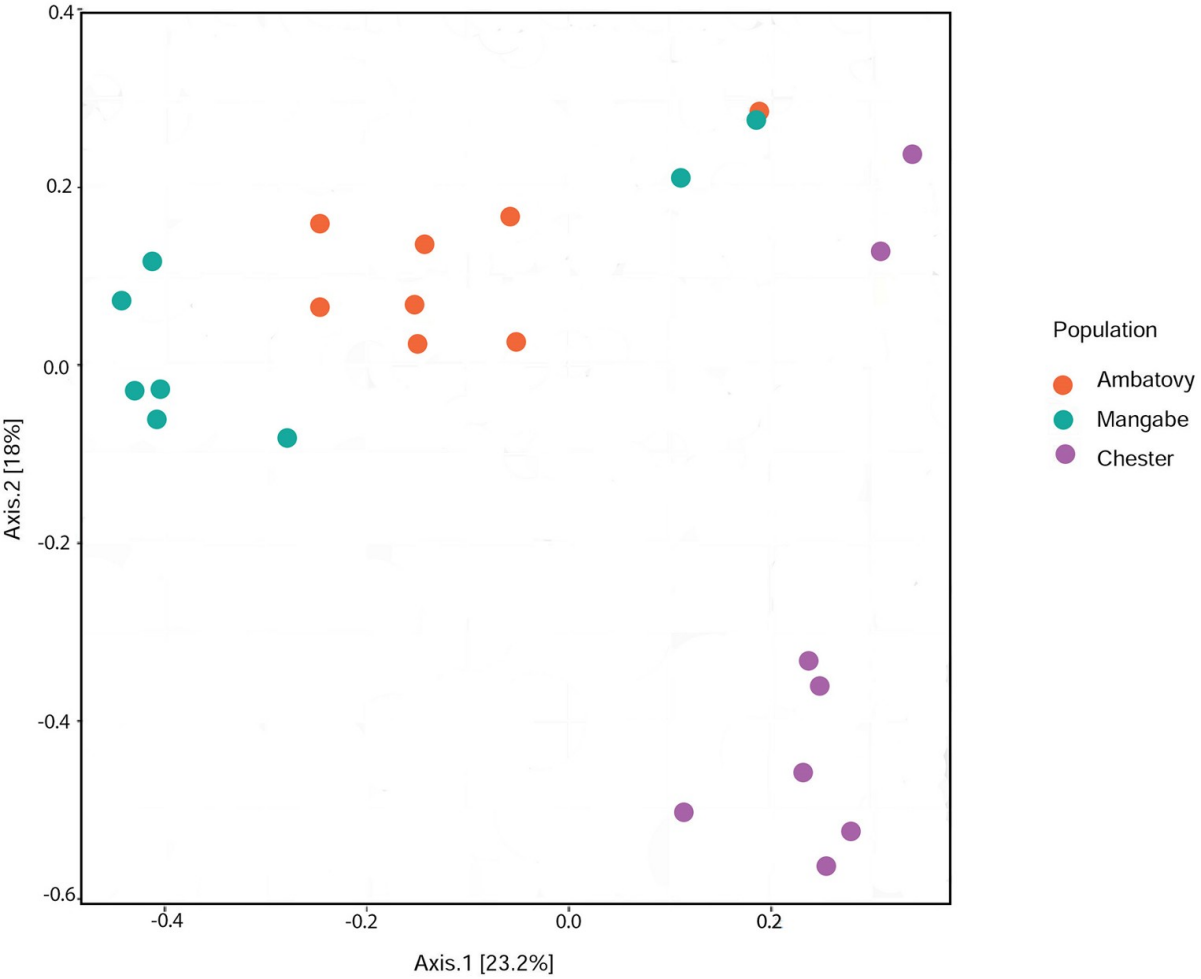
Plots from non-metric multidimensional scaling (nMDS) analyses representing the population-related differences in the composition of the skin bacterial communities of three populations of golden mantella frogs (Chester Zoo, Ambatovy and Mangabe).

The Adonis model showed that origin (wild versus captive) (Pseudo-F_1,22_ = 4.02, R^2^ = 7.20, df = 1, p<0.001) and population (i.e. Ambatovy, Chester Zoo and Mangabe) (Pseudo-F_2,22_ = 2.84, R^2^ = 7.71, df = 2, p<0.001) had a significant effect on the overall bacterial community composition associated with frogs (Fig 3). Neither the sex of frogs nor their body condition had a significant effect on bacterial composition when comparing wild and captive animals or in each population separately (p>0.05 in all cases).

Differential relative abundant analyses using DESeq2 on the unrarefied data set identified 209 OTUs that were more abundant in Mangabe, 90 that were more abundant in Ambatovy and only 5 that were more abundant in Chester.

## Discussion

During this study, we found that golden mantella frogs kept in captivity presented significantly different skin microbiota composition in comparison to wild conspecifics. This result was expected considering previous studies that also found similar results with captive colonies having a less rich and abundant skin-associated microbiota [5, 14, 15, 9, 32]. Given the important role symbiotic microbiota communities have for the innate immunity of the host amphibian [33], the findings of this study are important for conservation.

Skin bacterial communities of captive and wild golden mantella frogs were dominated by Gammaproteobacteria, and Actinobacteria, which is in agreement with findings from amphibian studies in North America [34, 35], Central America [36], Europe [37] and Japan [9]. All three populations showed a higher prevalence of the Enterobacteriaceae family, Chester Zoo’s animals also had a higher incidence of the Xanthomonadaceae and Sinobacteaceae, families that were not found on any of the wild populations. Ambatovy samples had the Plactomycetaceae as the second most common family, while Mangabe skin associated bacteria were distributed across different families, many of which were not presented in captive samples.

The composition of microbiota associated with amphibians’ skin is determined by a diversity of factors, and disentangling these is challenging [9]. The reduction of bacterial diversity in captivity may lead to a higher susceptibility of the frogs to diseases [38]. Therefore, this needs to be considered for *ex situ* management of threatened amphibians, especially in projects that have as the goal of reintroducing individuals to the wild [4].

Studies suggest that the structure of the microbial communities can have direct impacts on their function, and ultimately on host phenotype [39, 40]. Communities that are richer in species would have an increased ability to produce antifungal metabolites and, as a result, protect their hosts against infections [40,41]. Several studies have already provided evidence consistent with a correlation between overall microbiome diversity and susceptibility to infectious disease and costs associated with host responses to pathogen exposure [5]. A higher diversity of symbiont communities is linked with a stronger resistance to cutaneous infections [40]. The bacterial community observed on captive animals could be less efficient in protecting their host against pathogens due to its different and less rich composition.

Besides the differences observed on the microbiota found on wild and captive animals, it is important to discuss the similarities observed. All populations showed a high prevalence of the Enterobacteriaceae family, as it has been demonstrated for other species of frogs [42]. Some OTUs of the Enterobacteriaceae family (*Cedecea and Proteus*) that were observed on golden mantella samples are associated with soil and water [42]. These results would be in accordance with the hypothesis that frog microbiota is obtained through environmental sources and mediated through environmental factors.

Previous studies focusing on understanding the functionality of the microbiota derived from frogs’ skin have identified important genera for inhibition of pathogens growth that were also found in our samples, such as *Pseudomona*s, *Serratia*, *Enterobacter* and *Acinetobacter*, [7,35,42,43]. Chytrid inhibitory OTUs were also identified in ours samples [6, 44,45], such as *Acinetobacter*, *Chryseobacterium* and *Pseudomona*s on all three populations. While *Janthinobacterium* and *Pedobacter* were only found in samples from wild golden mantellas frogs. Despite being found in low abundance, it is important to emphasize the presence of these genera. If these bacteria occur naturally on the golden mantella frogs then wild individuals could, potentially, have a natural resistance to this fungus. This shows that even though captive frogs have a simpler bacterial composition on their skin, is it possible that this microbiota still retains its functionality against pathogens.

Microbiota reservoirs (e.g., water, soil, and plants) appear to be sources of skin microbiota for frogs, and host internal drivers (life stage, skin secretions) might help sculpt the composition of these communities [7, 9, 15, 32, 46]. Captive environments are less complex than wild environments and, are routinely cleaned by keepers, with water drained and substrate changed [16]. This could prevent bacteria colonies from developing and, consequently, associating with the frogs’ skin [12].

The results found here showed that even though the microbiota found on the skin of captive golden mantella frogs is much simpler than what was observed on wild individuals, it still seems to preserve some important strains of pathogen inhibitory bacteria. The next steps in this line of research should include investigating how the reintroduction of golden mantella frogs to their native habitat will likely affect their skin-associated microbial community.

The main concern about the species poor bacterial community on the skin of captive golden mantella frogs was related to the plans for reintroduction of captive bred individuals to the wild. The lack of some bacteria species could prevent individuals from being able to resist some natural pathogens in the wild [9]. Recent studies have already detected the presence of the amphibian chytrid fungus (Bd), in wild populations of amphibians in Madagascar, including regions near the golden mantella frog’s occurrence [13]. Releasing animals with lower survival chances would reduce the conservation value of a reintroduction and would be ethically questionable.

There are still many factors to be considered to understand the dynamics of amphibian skin associated bacterial communities, their composition and variation. Ongoing studies are trying to discover how to improve the host bacteria assemblage using probiotics [13, 19, 47]. More research is required to investigate how bacterial communities change over time (generations) when host organisms are brought into captivity, and how this may affect their susceptibility to disease [14]. Most available studies focus on the more abundant members of the bacterial communities, but future work on rare OTUs is necessary because these could have important roles for host health [5]. The development of methods to maintain and manipulate bacterial communities are fundamental for conservation management of captive and wild amphibian populations [9].

## Supporting information

S1 Appendix List of all Operational Taxonomic Units (OTUs) identified during the 16S Next Generation Sequencing in each of the sample populations (Mangabe (wild), Ambatovy (Wild) and Chester Zoo (captive)). (DOCX)
