# Supplementary material for: Comparing the bacterial communities of wild and captive golden mantella frogs: Implications for amphibian conservation
Source: PLoS One. 2018 Oct 31;13(10):e0205652. doi: 10.1371/journal.pone.0205652 (PMC6209184; doi:10.1371/journal.pone.0205652)
Supplement: S1 Appendix — (DOCX) [file pone.0205652.s001.docx]

Appendix 1. List of all Operational Taxonomic Units (OTUs) identified during the 16S Next Generation Sequencing in each of the sample populations (Mangabe (wild), Ambatovy (Wild) and Chester Zoo (captive)).

| **OTUid** | **Kingdom** | **Phylum** | **Class** | **Order** | **Family** | **Genus** | **Mangabe** | | **Ambatovy** | | | **Chester** | |
| --- | --- | --- | --- | --- | --- | --- | --- | --- | --- | --- | --- | --- | --- |
| OTU_1 | Bacteria | Proteobacteria | Gammaproteobacteria | Enterobacteriales | Enterobacteriaceae | Serratia | * | | * | | | * | |
| OTU_2 | Bacteria | Proteobacteria | Gammaproteobacteria | Enterobacteriales | Enterobacteriaceae | NA | * | | * | | | * | |
| OTU_3 | Bacteria | Proteobacteria | Gammaproteobacteria | Enterobacteriales | Enterobacteriaceae | Salmonella | * | | * | | | * | |
| OTU_4 | Bacteria | Proteobacteria | Gammaproteobacteria | Enterobacteriales | Enterobacteriaceae | Serratia | * | | * | | | * | |
| OTU_5 | Bacteria | Proteobacteria | Gammaproteobacteria | Enterobacteriales | Enterobacteriaceae | Enterobacter | * | |  | | | * | |
| OTU_6 | Bacteria | Actinobacteria | Actinobacteria | Actinomycetales | Microbacteriaceae | NA | * | | * | | | * | |
| OTU_7 | Bacteria | Proteobacteria | Gammaproteobacteria | Enterobacteriales | Enterobacteriaceae | Providencia | * | |  | | | * | |
| OTU_8 | Bacteria | Proteobacteria | Betaproteobacteria | Burkholderiales | Alcaligenaceae | Bordetella | * | |  | | | * | |
| OTU_9 | Bacteria | Proteobacteria | Gammaproteobacteria | Enterobacteriales | Enterobacteriaceae | NA |  | |  | | | * | |
| OTU_10 | Bacteria | Proteobacteria | Gammaproteobacteria | Enterobacteriales | Enterobacteriaceae | Proteus |  | |  | | | * | |
| OTU_11 | Bacteria | NA | NA | NA | NA | NA | * | |  | | |  | |
| OTU_12 | Bacteria | Proteobacteria | Alphaproteobacteria | Rhizobiales | Bradyrhizobiaceae | Bradyrhizobium | * | |  | | |  | |
| OTU_13 | Bacteria | Proteobacteria | Gammaproteobacteria | Pseudomonadales | Moraxellaceae | Enhydrobacter |  | | * | | | * | |
| OTU_14 | Bacteria | Proteobacteria | Gammaproteobacteria | Xanthomonadales | Xanthomonadaceae | Rhodanobacter |  | |  | | | * | |
| OTU_15 | Bacteria | Proteobacteria | Alphaproteobacteria | Sphingomonadales | Sphingomonadaceae | Sphingomonas | * | |  | | |  | |
| OTU_16 | Bacteria | Cyanobacteria | Chloroplast | Chloroplast | Bacillariophyta | NA |  | |  | | | * | |
| OTU_17 | Bacteria | Proteobacteria | Gammaproteobacteria | Oceanospirillales | Halomonadaceae | Halomonas |  | |  | | | * | |
| OTU_18 | Bacteria | Saccharibacteria | NA | NA | NA | NA |  | |  | | | * | |
| OTU_19 | Bacteria | Proteobacteria | Gammaproteobacteria | Pseudomonadales | Pseudomonadaceae | Pseudomonas |  | | * | | | * | |
| OTU_20 | Bacteria | Proteobacteria | Betaproteobacteria | Methylophilales | Methylophilaceae | Methylophilus |  | |  | | | * | |
| OTU_21 | Bacteria | Proteobacteria | Gammaproteobacteria | Xanthomonadales | Sinobacteraceae | Alkanibacter |  | |  | | | * | |
| OTU_22 | Bacteria | Cyanobacteria | Chloroplast | Chloroplast | Streptophyta | NA | * | |  | | | * | |
| OTU_23 | Bacteria | Proteobacteria | Alphaproteobacteria | Rhizobiales | NA | NA |  | | * | | |  | |
| OTU_24 | Bacteria | Proteobacteria | Alphaproteobacteria | Rhizobiales | Beijerinckiaceae | NA |  | | * | | |  | |
| OTU_25 | Bacteria | Proteobacteria | Betaproteobacteria | Burkholderiales | Alcaligenaceae | Candidimonas |  | |  | | | * | |
| OTU_26 | Bacteria | Proteobacteria | Alphaproteobacteria | Rhizobiales | NA | NA | * | |  | | |  | |
| OTU_27 | Bacteria | Proteobacteria | Gammaproteobacteria | Oceanospirillales | Alcanivoracaceae | Alcanivorax |  | |  | | | * | |
| OTU_28 | Bacteria | Proteobacteria | Gammaproteobacteria | Xanthomonadales | Xanthomonadaceae | Wohlfahrtiimonas |  | |  | | | * | |
| OTU_29 | Bacteria | Proteobacteria | Betaproteobacteria | Methylophilales | Methylophilaceae | Methylophilus |  | |  | | | * | |
| OTU_30 | Bacteria | Proteobacteria | Alphaproteobacteria | Alphaproteobacteria_incertae_sedis | Rhizomicrobium | NA | * | |  | | |  | |
| OTU_31 | Bacteria | Actinobacteria | Actinobacteria | Rubrobacterales | Rubrobacteraceae | Rubrobacter | * | |  | | |  | |
| OTU_32 | Bacteria | Actinobacteria | Actinobacteria | Actinomycetales | Brevibacteriaceae | Brevibacterium |  | | * | | | * | |
| OTU_33 | Bacteria | Proteobacteria | Gammaproteobacteria | Xanthomonadales | Sinobacteraceae | Alkanibacter |  | |  | | | * | |
| OTU_34 | Bacteria | Actinobacteria | Actinobacteria | Actinomycetales | Propionibacteriaceae | Propionibacterium | * | | * | | |  | |
| OTU_35 | Bacteria | Proteobacteria | NA | NA | NA | NA |  | |  | | | * | |
| OTU_36 | Bacteria | Proteobacteria | Betaproteobacteria | Burkholderiales | Burkholderiaceae | Burkholderia | * | | * | | |  | |
| OTU_37 | Bacteria | Saccharibacteria | NA | NA | NA | NA |  | | * | | |  | |
| OTU_38 | Bacteria | Proteobacteria | Gammaproteobacteria | Pseudomonadales | Pseudomonadaceae | Pseudomonas |  | | * | | |  | |
| OTU_39 | Bacteria | Proteobacteria | Gammaproteobacteria | Pseudomonadales | Moraxellaceae | Alkanindiges |  | | * | | |  | |
| OTU_40 | Bacteria | Firmicutes | Bacilli | Bacillales | Staphylococcaceae | Staphylococcus | * | | * | | |  | |
| OTU_41 | Bacteria | Proteobacteria | Alphaproteobacteria | Rhizobiales | Methylobacteriaceae | Methylobacterium | * | | * | | |  | |
| OTU_42 | Bacteria | Proteobacteria | Betaproteobacteria | Burkholderiales | NA | NA |  | |  | | | * | |
| OTU_43 | Bacteria | Proteobacteria | Alphaproteobacteria | Rhizobiales | Beijerinckiaceae | NA | * | |  | | |  | |
| OTU_44 | Bacteria | Actinobacteria | Actinobacteria | Actinomycetales | Microbacteriaceae | Microbacterium |  | | * | | |  | |
| OTU_45 | Bacteria | Acidobacteria | Acidobacteria_Gp4 | Blastocatella | NA | NA |  | | * | | |  | |
| OTU_46 | Bacteria | Actinobacteria | Actinobacteria | Solirubrobacterales | Conexibacteraceae | Conexibacter | * | |  | | |  | |
| OTU_47 | Bacteria | Proteobacteria | Alphaproteobacteria | Rhizobiales | Aurantimonadaceae | Aurantimonas |  | |  | | | * | |
| OTU_48 | Bacteria | Proteobacteria | Alphaproteobacteria | Rhizobiales | Beijerinckiaceae | Beijerinckia | * | |  | | |  | |
| OTU_49 | Bacteria | Planctomycetes | Planctomycetia | Planctomycetales | Planctomycetaceae | Singulisphaera | * | |  | | |  | |
| OTU_50 | Bacteria | Proteobacteria | Gammaproteobacteria | Xanthomonadales | Xanthomonadaceae | NA |  | |  | | | * | |
| OTU_51 | Bacteria | Proteobacteria | Gammaproteobacteria | Xanthomonadales | Xanthomonadaceae | Luteibacter |  | |  | | | * | |
| OTU_52 | Bacteria | Proteobacteria | Betaproteobacteria | Burkholderiales | Comamonadaceae | Acidovorax | * | |  | | |  | |
| OTU_53 | Bacteria | Proteobacteria | Betaproteobacteria | Burkholderiales | Burkholderiaceae | Burkholderia | * | |  | | |  | |
| OTU_54 | Bacteria | Proteobacteria | Gammaproteobacteria | Pseudomonadales | Pseudomonadaceae | Rhizobacter | * | | * | | |  | |
| OTU_55 | Bacteria | Proteobacteria | Gammaproteobacteria | Xanthomonadales | Xanthomonadaceae | Arenimonas | * | |  | | |  | |
| OTU_56 | Bacteria | Proteobacteria | Betaproteobacteria | Burkholderiales | Comamonadaceae | Variovorax | * | |  | | |  | |
| OTU_57 | Bacteria | Verrucomicrobia | Spartobacteria | NA | NA | NA |  | | * | | |  | |
| OTU_58 | Bacteria | Proteobacteria | Alphaproteobacteria | Rhizobiales | Bradyrhizobiaceae | Bradyrhizobium |  | | * | | |  | |
| OTU_59 | Bacteria | Proteobacteria | Gammaproteobacteria | Pseudomonadales | Pseudomonadaceae | Pseudomonas | * | |  | | |  | |
| OTU_60 | Bacteria | Acidobacteria | Acidobacteria_Gp2 | Gp2 | NA | NA | * | | * | | |  | |
| OTU_61 | Bacteria | Proteobacteria | Betaproteobacteria | Burkholderiales | Comamonadaceae | NA | * | |  | | |  | |
| OTU_62 | Bacteria | Proteobacteria | Alphaproteobacteria | Rhodospirillales | NA | NA |  | | * | | |  | |
| OTU_63 | Bacteria | Actinobacteria | Actinobacteria | Acidimicrobiales | Iamiaceae | Iamia | * | | * | | |  | |
| OTU_64 | Bacteria | Firmicutes | Clostridia | Clostridiales | Lachnospiraceae | NA |  | | * | | |  | |
| OTU_65 | Bacteria | Proteobacteria | Alphaproteobacteria | Rhizobiales | NA | NA |  | | * | | |  | |
| OTU_66 | Bacteria | Acidobacteria | Acidobacteria_Gp1 | Gp1 | NA | NA |  | | * | | |  | |
| OTU_67 | Bacteria | Actinobacteria | Actinobacteria | Actinomycetales | Pseudonocardiaceae | Pseudonocardia |  | |  | | | * | |
| OTU_68 | Bacteria | Proteobacteria | Gammaproteobacteria | Xanthomonadales | Xanthomonadaceae | Rhodanobacter |  | |  | | | * | |
| OTU_69 | Bacteria | Verrucomicrobia | Spartobacteria | NA | NA | NA |  | | * | | |  | |
| OTU_70 | Bacteria | Actinobacteria | Actinobacteria | Actinomycetales | Micrococcaceae | Micrococcus |  | | * | | |  | |
| OTU_71 | Bacteria | Actinobacteria | Actinobacteria | Actinomycetales | Dermacoccaceae | NA |  | |  | | | * | |
| OTU_72 | Bacteria | Actinobacteria | Actinobacteria | Actinomycetales | Mycobacteriaceae | Mycobacterium |  | | * | | |  | |
| OTU_73 | Bacteria | Planctomycetes | Planctomycetia | Planctomycetales | Planctomycetaceae | Aquisphaera |  | | * | | |  | |
| OTU_74 | Bacteria | Bacteroidetes | Flavobacteriia | Flavobacteriales | Flavobacteriaceae | Myroides |  | |  | | | * | |
| OTU_75 | Bacteria | Bacteroidetes | Sphingobacteriia | Sphingobacteriales | Sphingobacteriaceae | Mucilaginibacter | * | |  | | |  | |
| OTU_76 | Bacteria | Actinobacteria | Actinobacteria | Actinomycetales | Nocardioidaceae | Aeromicrobium |  | | * | | |  | |
| OTU_77 | Bacteria | NA | NA | NA | NA | NA |  | | * | | |  | |
| OTU_78 | Bacteria | Acidobacteria | Acidobacteria_Gp1 | Gp1 | NA | NA |  | | * | | |  | |
| OTU_79 | Bacteria | Acidobacteria | Acidobacteria_Gp1 | Acidipila | NA | NA |  | | * | | |  | |
| OTU_80 | Bacteria | Proteobacteria | Gammaproteobacteria | Pseudomonadales | Moraxellaceae | Acinetobacter |  | | * | | |  | |
| OTU_81 | Bacteria | Actinobacteria | Actinobacteria | Actinomycetales | Micromonosporaceae | Actinoplanes | * | |  | | |  | |
| OTU_82 | Bacteria | Proteobacteria | Epsilonproteobacteria | Campylobacterales | Helicobacteraceae | Helicobacter |  | | * | | |  | |
| OTU_83 | Bacteria | Verrucomicrobia | Spartobacteria | NA | NA | NA |  | | * | | |  | |
| OTU_84 | Bacteria | Planctomycetes | Planctomycetia | Planctomycetales | Planctomycetaceae | Aquisphaera |  | | * | | |  | |
| OTU_85 | Bacteria | Proteobacteria | Betaproteobacteria | Burkholderiales | Oxalobacteraceae | Janthinobacterium |  | | * | | |  | |
| OTU_86 | Bacteria | Firmicutes | Clostridia | Clostridiales | Lachnospiraceae | NA |  | | * | | |  | |
| OTU_87 | Bacteria | Proteobacteria | Gammaproteobacteria | Oceanospirillales | Alcanivoracaceae | Alcanivorax |  | |  | | | * | |
| OTU_88 | Bacteria | Acidobacteria | Acidobacteria_Gp2 | Gp2 | NA | NA | * | | * | | |  | |
| OTU_89 | Bacteria | Actinobacteria | Actinobacteria | Actinomycetales | Mycobacteriaceae | Mycobacterium |  | |  | | | * | |
| OTU_90 | Bacteria | Acidobacteria | Acidobacteria_Gp1 | Gp1 | NA | NA | * | |  | | |  | |
| OTU_91 | Bacteria | Planctomycetes | Planctomycetia | Planctomycetales | Planctomycetaceae | Aquisphaera |  | | * | | |  | |
| OTU_92 | Bacteria | Deinococcus-Thermus | Deinococci | Deinococcales | Deinococcaceae | Deinococcus |  | | * | | |  | |
| OTU_93 | Bacteria | Proteobacteria | Betaproteobacteria | Burkholderiales | Burkholderiaceae | Burkholderia |  | | * | | |  | |
| OTU_94 | Bacteria | Proteobacteria | NA | NA | NA | NA | * | |  | | |  | |
| OTU_95 | Bacteria | Bacteroidetes | Sphingobacteriia | Sphingobacteriales | Chitinophagaceae | NA | * | |  | | |  | |
| OTU_96 | Bacteria | Proteobacteria | Alphaproteobacteria | Rhizobiales | Beijerinckiaceae | Methylovirgula | * | |  | | |  | |
| OTU_97 | Bacteria | Acidobacteria | Acidobacteria_Gp1 | Granulicella | NA | NA | * | |  | | |  | |
| OTU_98 | Bacteria | Actinobacteria | Actinobacteria | Actinomycetales | Corynebacteriaceae | Corynebacterium |  | | * | | |  | |
| OTU_99 | Bacteria | Proteobacteria | Gammaproteobacteria | Xanthomonadales | Sinobacteraceae | Alkanibacter |  | |  | | | * | |
| OTU_100 | Bacteria | Proteobacteria | Gammaproteobacteria | Xanthomonadales | Xanthomonadaceae | Luteibacter |  | |  | | | * | |
| OTU_101 | Bacteria | Bacteroidetes | Flavobacteriia | Flavobacteriales | Cryomorphaceae | Fluviicola | * | |  | | |  | |
| OTU_102 | Bacteria | Actinobacteria | Actinobacteria | Actinomycetales | Mycobacteriaceae | Mycobacterium |  | | * | | |  | |
| OTU_103 | Bacteria | Planctomycetes | Planctomycetia | Planctomycetales | Planctomycetaceae | NA |  | | * | | |  | |
| OTU_104 | Bacteria | Proteobacteria | Betaproteobacteria | Burkholderiales | Burkholderiaceae | Ralstonia |  | | * | | |  | |
| OTU_105 | Bacteria | Bacteroidetes | Sphingobacteriia | Sphingobacteriales | Chitinophagaceae | Flavisolibacter |  | | * | | |  | |
| OTU_106 | Bacteria | Proteobacteria | Alphaproteobacteria | Rhizobiales | Rhizobiaceae | Rhizobium |  | | * | | |  | |
| OTU_107 | Bacteria | Actinobacteria | Actinobacteria | Actinomycetales | Corynebacteriaceae | Corynebacterium |  | | * | | |  | |
| OTU_108 | Bacteria | Proteobacteria | Alphaproteobacteria | Sphingomonadales | Sphingomonadaceae | Sphingomonas |  | |  | | | * | |
| OTU_109 | Bacteria | Firmicutes | Bacilli | Lactobacillales | Enterococcaceae | Vagococcus |  | |  | | | * | |
| OTU_110 | Bacteria | Actinobacteria | Actinobacteria | Actinomycetales | NA | NA |  | |  | | | * | |
| OTU_111 | Bacteria | Proteobacteria | Alphaproteobacteria | Rhizobiales | Hyphomicrobiaceae | Devosia | * | |  | | |  | |
| OTU_112 | Bacteria | Verrucomicrobia | Spartobacteria | NA | NA | NA | * | |  | | |  | |
| OTU_113 | Bacteria | NA | NA | NA | NA | NA |  | | * | | |  | |
| OTU_114 | Bacteria | Proteobacteria | Gammaproteobacteria | NA | NA | NA |  | | * | | |  | |
| OTU_115 | Bacteria | Proteobacteria | Gammaproteobacteria | Xanthomonadales | Xanthomonadaceae | Luteibacter |  | |  | | | * | |
| OTU_116 | Bacteria | Proteobacteria | Alphaproteobacteria | Sphingomonadales | Sphingomonadaceae | Sphingomonas |  | | * | | |  | |
| OTU_117 | Bacteria | Verrucomicrobia | Subdivision3 | NA | NA | NA |  | | * | | |  | |
| OTU_118 | Bacteria | NA | NA | NA | NA | NA |  | | * | | |  | |
| OTU_119 | Bacteria | Proteobacteria | Gammaproteobacteria | Pseudomonadales | Moraxellaceae | Acinetobacter |  | | * | | |  | |
| OTU_120 | Bacteria | NA | NA | NA | NA | NA |  | | * | | |  | |
| OTU_121 | Bacteria | Actinobacteria | Actinobacteria | Actinomycetales | Mycobacteriaceae | Mycobacterium | * | |  | | |  | |
| OTU_122 | Bacteria | Acidobacteria | Acidobacteria_Gp1 | Granulicella | NA | NA | * | |  | | |  | |
| OTU_123 | Bacteria | Proteobacteria | Gammaproteobacteria | Enterobacteriales | Enterobacteriaceae | Citrobacter | * | |  | | |  | |
| OTU_124 | Bacteria | Bacteroidetes | Cytophagia | Cytophagales | Cytophagaceae | Spirosoma |  | | * | | |  | |
| OTU_125 | Bacteria | Bacteroidetes | Flavobacteriia | Flavobacteriales | Flavobacteriaceae | Chryseobacterium |  | |  | | | * | |
| OTU_126 | Bacteria | Proteobacteria | Alphaproteobacteria | Rhizobiales | Hyphomicrobiaceae | Devosia | * | |  | | |  | |
| OTU_127 | Bacteria | Planctomycetes | Planctomycetia | Planctomycetales | Planctomycetaceae | Planctomyces | * | |  | | |  | |
| OTU_128 | NA | NA | NA | NA | NA | NA | * | |  | | |  | |
| OTU_129 | Bacteria | Acidobacteria | Acidobacteria_Gp1 | Gp1 | NA | NA |  | | * | | |  | |
| OTU_130 | Bacteria | Bacteroidetes | Bacteroidia | Bacteroidales | NA | NA |  | | * | | |  | |
| OTU_131 | Bacteria | Acidobacteria | Acidobacteria_Gp1 | Gp1 | NA | NA |  | | * | | |  | |
| OTU_132 | Bacteria | Proteobacteria | Deltaproteobacteria | Myxococcales | NA | NA |  | | * | | |  | |
| OTU_133 | Bacteria | Firmicutes | Bacilli | Bacillales | Bacillaceae_1 | Bacillus |  | | * | | |  | |
| OTU_134 | Bacteria | Actinobacteria | Actinobacteria | Solirubrobacterales | Conexibacteraceae | Conexibacter |  | |  | | | * | |
| OTU_135 | Bacteria | Proteobacteria | Gammaproteobacteria | Pseudomonadales | Pseudomonadaceae | Pseudomonas | * | |  | | |  | |
| OTU_136 | Bacteria | Bacteroidetes | Sphingobacteriia | Sphingobacteriales | Sphingobacteriaceae | Sphingobacterium |  | | * | | |  | |
| OTU_137 | Bacteria | candidate_divisio | NA | NA | NA | NA |  | | * | | |  | |
| OTU_138 | Bacteria | Proteobacteria | Betaproteobacteria | Burkholderiales | Burkholderiaceae | Burkholderia |  | | * | | |  | |
| OTU_139 | Bacteria | Actinobacteria | Actinobacteria | Actinomycetales | Dermacoccaceae | Branchiibius |  | | * | | |  | |
| OTU_140 | Bacteria | Verrucomicrobia | Subdivision3 | NA | NA | NA |  | | * | | |  | |
| OTU_141 | Bacteria | NA | NA | NA | NA | NA |  | | * | | |  | |
| OTU_142 | Bacteria | Acidobacteria | Acidobacteria_Gp1 | Acidobacterium | NA | NA |  | | * | | |  | |
| OTU_143 | Bacteria | Actinobacteria | Actinobacteria | Actinomycetales | Pseudonocardiaceae | Actinomycetospora |  | |  | | | * | |
| OTU_144 | Bacteria | Saccharibacteria | NA | NA | NA | NA |  | |  | | | * | |
| OTU_145 | Bacteria | Proteobacteria | Betaproteobacteria | Burkholderiales | Burkholderiaceae | Cupriavidus |  | |  | | | * | |
| OTU_146 | Bacteria | Proteobacteria | Alphaproteobacteria | Rhizobiales | Brucellaceae | Pseudochrobactrum | * | |  | | |  | |
| OTU_147 | Bacteria | Planctomycetes | Planctomycetia | Planctomycetales | Planctomycetaceae | Blastopirellula |  | | * | | |  | |
| OTU_148 | Bacteria | Proteobacteria | Alphaproteobacteria | Caulobacterales | Caulobacteraceae | NA |  | | * | | |  | |
| OTU_149 | Bacteria | Proteobacteria | Alphaproteobacteria | Rhodospirillales | Acetobacteraceae | Acidisoma |  | | * | | |  | |
| OTU_150 | Bacteria | Actinobacteria | Actinobacteria | Actinomycetales | Mycobacteriaceae | Mycobacterium |  | | * | | |  | |
| OTU_151 | Bacteria | Proteobacteria | Gammaproteobacteria | Chromatiales | Ectothiorhodospiraceae | NA |  | | * | | |  | |
| OTU_152 | Bacteria | Planctomycetes | Planctomycetia | Planctomycetales | Planctomycetaceae | NA |  | | * | | |  | |
| OTU_153 | Bacteria | Proteobacteria | Gammaproteobacteria | Xanthomonadales | Xanthomonadaceae | NA |  | |  | | | * | |
| OTU_154 | Bacteria | Proteobacteria | Deltaproteobacteria | Myxococcales | Polyangiaceae | Sorangium | * | |  | | |  | |
| OTU_155 | Bacteria | Bacteroidetes | Sphingobacteriia | Sphingobacteriales | Chitinophagaceae | NA |  | | * | | |  | |
| OTU_156 | Bacteria | Firmicutes | Bacilli | Bacillales | Staphylococcaceae | Staphylococcus |  | | * | | |  | |
| OTU_157 | Bacteria | Planctomycetes | Planctomycetia | Planctomycetales | Planctomycetaceae | Planctomyces |  | | * | | |  | |
| OTU_158 | Bacteria | Proteobacteria | Gammaproteobacteria | Chromatiales | Ectothiorhodospiraceae | NA |  | | * | | |  | |
| OTU_159 | Bacteria | Actinobacteria | Actinobacteria | Solirubrobacterales | Conexibacteraceae | Conexibacter |  | | * | | |  | |
| OTU_160 | Bacteria | NA | NA | NA | NA | NA |  | | * | | |  | |
| OTU_161 | Bacteria | Proteobacteria | Alphaproteobacteria | Rhizobiales | Phyllobacteriaceae | Mesorhizobium |  | |  | | | * | |
| OTU_162 | Bacteria | Proteobacteria | Alphaproteobacteria | Rhizobiales | Bradyrhizobiaceae | Bosea | * | |  | | |  | |
| OTU_163 | Bacteria | Proteobacteria | Gammaproteobacteria | Xanthomonadales | Xanthomonadaceae | NA | * | |  | | |  | |
| OTU_164 | Bacteria | Bacteroidetes | Flavobacteriia | Flavobacteriales | Flavobacteriaceae | Chryseobacterium | * | |  | | |  | |
| OTU_165 | Bacteria | Proteobacteria | Alphaproteobacteria | Sphingomonadales | Sphingomonadaceae | Novosphingobium | * | |  | | |  | |
| OTU_166 | Bacteria | Candidatus_Saccharibacteria | NA | NA | NA | NA |  | | * | | |  | |
| OTU_167 | Bacteria | Proteobacteria | Alphaproteobacteria | Alphaproteobacteria_incertae_sedis | Rhizomicrobium | NA |  | | * | | |  | |
| OTU_168 | Bacteria | Planctomycetes | Planctomycetia | Planctomycetales | Planctomycetaceae | Singulisphaera |  | | * | | |  | |
| OTU_169 | Bacteria | division_WPS-1 | NA | NA | NA | NA |  | | * | | |  | |
| OTU_170 | Bacteria | NA | NA | NA | NA | NA |  | | * | | |  | |
| OTU_171 | Bacteria | NA | NA | NA | NA | NA |  | | * | | |  | |
| OTU_172 | Bacteria | Proteobacteria | Alphaproteobacteria | Sphingomonadales | Erythrobacteraceae | NA |  | |  | | | * | |
| OTU_173 | Bacteria | Proteobacteria | Gammaproteobacteria | Pseudomonadales | Moraxellaceae | Acinetobacter |  | |  | | | * | |
| OTU_174 | Bacteria | Proteobacteria | Gammaproteobacteria | Pseudomonadales | Moraxellaceae | Acinetobacter |  | |  | | | * | |
| OTU_175 | Bacteria | Proteobacteria | Deltaproteobacteria | Myxococcales | Haliangiaceae | Haliangium | * | |  | | |  | |
| OTU_176 | Bacteria | Proteobacteria | Alphaproteobacteria | Rhizobiales | Bradyrhizobiaceae | Afipia |  | | * | | |  | |
| OTU_177 | Bacteria | Planctomycetes | Planctomycetia | Planctomycetales | Planctomycetaceae | NA |  | | * | | |  | |
| OTU_178 | Bacteria | Actinobacteria | Actinobacteria | Actinomycetales | Thermomonosporaceae | NA |  | | * | | |  | |
| OTU_179 | Bacteria | Acidobacteria | Acidobacteria_Gp3 | Candidatus_Solibacter | NA | NA |  | | * | | |  | |
| OTU_180 | Bacteria | Actinobacteria | Actinobacteria | Actinomycetales | Geodermatophilaceae | NA |  | | * | | |  | |
| OTU_181 | Bacteria | Actinobacteria | Actinobacteria | Actinomycetales | Nocardioidaceae | Marmoricola |  | |  | | | * | |
| OTU_182 | Bacteria | Actinobacteria | Actinobacteria | Actinomycetales | Nocardiaceae | Rhodococcus |  | |  | | | * | |
| OTU_183 | Bacteria | Proteobacteria | Gammaproteobacteria | Xanthomonadales | Xanthomonadaceae | Luteibacter | * | |  | | |  | |
| OTU_184 | Bacteria | Proteobacteria | Alphaproteobacteria | Rhodospirillales | Acetobacteraceae | Acidisoma | * | |  | | |  | |
| OTU_185 | Bacteria | Proteobacteria | Alphaproteobacteria | Rhodospirillales | Acetobacteraceae | NA | * | |  | | |  | |
| OTU_186 | Bacteria | Proteobacteria | Alphaproteobacteria | Rhizobiales | Methylobacteriaceae | Methylobacterium | * | |  | | |  | |
| OTU_187 | Bacteria | Bacteroidetes | Bacteroidetes_incertae_sedis | Ohtaekwangia | NA | NA | * | |  | | |  | |
| OTU_188 | Bacteria | Proteobacteria | Gammaproteobacteria | NA | NA | NA | * | |  | | |  | |
| OTU_189 | Bacteria | Planctomycetes | Planctomycetia | Planctomycetales | Planctomycetaceae | NA |  | | * | | |  | |
| OTU_190 | Bacteria | Planctomycetes | Planctomycetia | Planctomycetales | Planctomycetaceae | Aquisphaera |  | | * | | |  | |
| OTU_191 | Bacteria | Proteobacteria | NA | NA | NA | NA |  | | * | | |  | |
| OTU_192 | Bacteria | Actinobacteria | Actinobacteria | Actinomycetales | NA | NA |  | | * | | |  | |
| OTU_193 | Bacteria | Actinobacteria | Actinobacteria | Actinomycetales | NA | NA |  | | * | | |  | |
| OTU_194 | Bacteria | Planctomycetes | Planctomycetia | Planctomycetales | Planctomycetaceae | NA |  | | * | | |  | |
| OTU_195 | Bacteria | Acidobacteria | Acidobacteria_Gp1 | Terriglobus | NA | NA |  | | * | | |  | |
| OTU_196 | Bacteria | Proteobacteria | Betaproteobacteria | Burkholderiales | Burkholderiaceae | NA |  | | * | | |  | |
| OTU_197 | Bacteria | Actinobacteria | Actinobacteria | NA | NA | NA |  | | * | | |  | |
| OTU_198 | Bacteria | NA | NA | NA | NA | NA |  | | * | | |  | |
| OTU_199 | Bacteria | Bacteroidetes | Sphingobacteriia | Sphingobacteriales | Chitinophagaceae | NA |  | |  | | | * | |
| OTU_200 | Bacteria | Proteobacteria | Gammaproteobacteria | Xanthomonadales | Sinobacteraceae | Alkanibacter |  | |  | | | * | |
| OTU_201 | Bacteria | Bacteroidetes | Flavobacteriia | Flavobacteriales | Flavobacteriaceae | Myroides |  | |  | | | * | |
| OTU_202 | Bacteria | Proteobacteria | Gammaproteobacteria | NA | NA | NA | * | |  | | |  | |
| OTU_203 | Bacteria | Bacteroidetes | Cytophagia | Cytophagales | Cytophagaceae | Spirosoma | * | |  | | |  | |
| OTU_204 | Bacteria | Acidobacteria | Acidobacteria_Gp1 | Terriglobus | NA | NA | * | |  | | |  | |
| OTU_205 | Bacteria | Planctomycetes | Planctomycetia | Planctomycetales | Planctomycetaceae | Blastopirellula | * | |  | | |  | |
| OTU_206 | Bacteria | Bacteroidetes | Flavobacteriia | Flavobacteriales | Flavobacteriaceae | Flavobacterium | * | |  | | |  | |
| OTU_207 | Bacteria | Actinobacteria | Actinobacteria | Actinomycetales | Thermomonosporaceae | NA | * | |  | | |  | |
| OTU_208 | Bacteria | Proteobacteria | Deltaproteobacteria | Bdellovibrionales | Bdellovibrionaceae | Vampirovibrio |  | | * | | |  | |
| OTU_209 | Bacteria | Acidobacteria | Acidobacteria_Gp1 | Gp1 | NA | NA |  | | * | | |  | |
| OTU_210 | Bacteria | Verrucomicrobia | Subdivision3 | NA | NA | NA |  | | * | | |  | |
| OTU_211 | Bacteria | Firmicutes | Clostridia | Clostridiales | NA | NA |  | | * | | |  | |
| OTU_212 | Bacteria | Proteobacteria | Gammaproteobacteria | NA | NA | NA |  | | * | | |  | |
| OTU_213 | Bacteria | Bacteroidetes | Flavobacteriia | Flavobacteriales | Cryomorphaceae | NA |  | |  | | | * | |
| OTU_214 | Bacteria | Proteobacteria | Gammaproteobacteria | Xanthomonadales | Xanthomonadaceae | Luteibacter |  | |  | | | * | |
| OTU_215 | Bacteria | Proteobacteria | Gammaproteobacteria | Xanthomonadales | Sinobacteraceae | Nevskia |  | |  | | | * | |
| OTU_216 | Bacteria | Proteobacteria | Alphaproteobacteria | Sphingomonadales | Sphingomonadaceae | Sphingomonas | * | |  | | |  | |
| OTU_217 | Bacteria | Bacteroidetes | Sphingobacteriia | Sphingobacteriales | Chitinophagaceae | Terrimonas | * | |  | | |  | |
| OTU_218 | Bacteria | NA | NA | NA | NA | NA | * | |  | | |  | |
| OTU_219 | Bacteria | NA | NA | NA | NA | NA | * | |  | | |  | |
| OTU_220 | Bacteria | Verrucomicrobia | Subdivision3 | NA | NA | NA |  | | * | | |  | |
| OTU_221 | Bacteria | Planctomycetes | Planctomycetia | Planctomycetales | Planctomycetaceae | Singulisphaera |  | | * | | |  | |
| OTU_222 | Bacteria | Planctomycetes | Planctomycetia | Planctomycetales | Planctomycetaceae | NA |  | | * | | |  | |
| OTU_223 | Bacteria | Verrucomicrobia | Spartobacteria | NA | NA | NA |  | | * | | |  | |
| OTU_224 | Bacteria | Proteobacteria | Betaproteobacteria | Burkholderiales | Comamonadaceae | Pelomonas |  | | * | | |  | |
| OTU_225 | Bacteria | Actinobacteria | Actinobacteria | Actinomycetales | NA | NA |  | | * | | |  | |
| OTU_226 | Bacteria | Planctomycetes | Planctomycetia | Planctomycetales | Planctomycetaceae | Aquisphaera |  | | * | | |  | |
| OTU_227 | Bacteria | Proteobacteria | Alphaproteobacteria | Rhizobiales | Beijerinckiaceae | NA |  | | * | | |  | |
| OTU_228 | Bacteria | Proteobacteria | Gammaproteobacteria | Pseudomonadales | Moraxellaceae | Moraxella |  | | * | | |  | |
| OTU_229 | Bacteria | Proteobacteria | Betaproteobacteria | Burkholderiales | Comamonadaceae | Variovorax |  | | * | | |  | |
| OTU_230 | Bacteria | Planctomycetes | Planctomycetia | Planctomycetales | Planctomycetaceae | Aquisphaera |  | | * | | |  | |
| OTU_231 | Bacteria | Verrucomicrobia | NA | NA | NA | NA |  | | * | | |  | |
| OTU_232 | Archaea | Thaumarchaeota | Nitrososphaerales | Nitrososphaeraceae | Nitrososphaera | NA |  | | * | | |  | |
| OTU_233 | Bacteria | Proteobacteria | Deltaproteobacteria | Myxococcales | Polyangiaceae | NA |  | | * | | |  | |
| OTU_234 | Bacteria | Planctomycetes | Planctomycetia | Planctomycetales | Planctomycetaceae | Gemmata |  | | * | | |  | |
| OTU_235 | Bacteria | Acidobacteria | Acidobacteria_Gp1 | Gp1 | NA | NA |  | | * | | |  | |
| OTU_236 | Bacteria | Bacteroidetes | Sphingobacteriia | Sphingobacteriales | Chitinophagaceae | Flavihumibacter |  | |  | | | * | |
| OTU_237 | Bacteria | NA | NA | NA | NA | NA |  | |  | | | * | |
| OTU_238 | Bacteria | Saccharibacteria | NA | NA | NA | NA |  | |  | | | * | |
| OTU_239 | Bacteria | Proteobacteria | Gammaproteobacteria | Enterobacteriales | Enterobacteriaceae | NA | * | |  | | |  | |
| OTU_240 | Bacteria | Proteobacteria | Alphaproteobacteria | Alphaproteobacteria_incertae_sedis | Rhizomicrobium | NA | * | |  | | |  | |
| OTU_241 | Bacteria | Proteobacteria | Alphaproteobacteria | Caulobacterales | Caulobacteraceae | Caulobacter | * | |  | | |  | |
| OTU_242 | Bacteria | Bacteroidetes | Sphingobacteriia | Sphingobacteriales | Chitinophagaceae | NA | * | |  | | |  | |
| OTU_243 | Bacteria | Proteobacteria | Alphaproteobacteria | Rhizobiales | NA | NA | * | |  | | |  | |
| OTU_244 | Bacteria | Bacteroidetes | Sphingobacteriia | Sphingobacteriales | Chitinophagaceae | Hydrotalea |  | | * | | |  | |
| OTU_245 | Bacteria | Bacteroidetes | Sphingobacteriia | Sphingobacteriales | Sphingobacteriaceae | Mucilaginibacter |  | | * | | |  | |
| OTU_246 | Bacteria | Cyanobacteria | Chloroplast | Chloroplast | Streptophyta | NA |  | | * | | |  | |
| OTU_247 | Bacteria | Planctomycetes | Planctomycetia | Planctomycetales | Planctomycetaceae | Aquisphaera |  | | * | | |  | |
| OTU_248 | Bacteria | division_WPS-1 | NA | NA | NA | NA |  | | * | | |  | |
| OTU_249 | Bacteria | Bacteroidetes | Flavobacteriia | Flavobacteriales | Flavobacteriaceae | Chryseobacterium |  | | * | | |  | |
| OTU_250 | Bacteria | Acidobacteria | Acidobacteria_Gp2 | Gp2 | NA | NA |  | | * | | |  | |
| OTU_251 | Bacteria | Proteobacteria | Alphaproteobacteria | Rhodospirillales | Acetobacteraceae | Acidisoma |  | | * | | |  | |
| OTU_252 | Bacteria | NA | NA | NA | NA | NA |  | | * | | |  | |
| OTU_253 | Bacteria | Proteobacteria | Deltaproteobacteria | Myxococcales | Cystobacteraceae | NA |  | | * | | |  | |
| OTU_254 | Bacteria | Bacteroidetes | Sphingobacteriia | Sphingobacteriales | Sphingobacteriaceae | Sphingobacterium |  | | * | | | * | |
| OTU_255 | Bacteria | Actinobacteria | Actinobacteria | Actinomycetales | Nocardioidaceae | Nocardioides |  | |  | | | * | |
| OTU_256 | Bacteria | Proteobacteria | Alphaproteobacteria | Sphingomonadales | Sphingomonadaceae | Sphingomonas |  | |  | | | * | |
| OTU_257 | Bacteria | Armatimonadetes | Armatimonadetes_gp5 | NA | NA | NA |  | |  | | | * | |
| OTU_258 | Bacteria | Acidobacteria | Acidobacteria_Gp1 | Edaphobacter | NA | NA | * | |  | | |  | |
| OTU_259 | Bacteria | Bacteroidetes | NA | NA | NA | NA | * | |  | | |  | |
| OTU_260 | Bacteria | Proteobacteria | Deltaproteobacteria | Myxococcales | Haliangiaceae | Haliangium | * | |  | | |  | |
| OTU_261 | Bacteria | NA | NA | NA | NA | NA |  | | * | | |  | |
| OTU_262 | Bacteria | Proteobacteria | NA | NA | NA | NA |  | | * | | |  | |
| OTU_263 | Bacteria | Proteobacteria | Alphaproteobacteria | Caulobacterales | Caulobacteraceae | Phenylobacterium |  | | * | | |  | |
| OTU_264 | Bacteria | Proteobacteria | Alphaproteobacteria | Rhodospirillales | Acetobacteraceae | NA |  | | * | | |  | |
| OTU_265 | Bacteria | Proteobacteria | Alphaproteobacteria | NA | NA | NA |  | | * | | |  | |
| OTU_266 | Bacteria | Verrucomicrobia | Subdivision3 | NA | NA | NA |  | | * | | |  | |
| OTU_267 | Bacteria | Planctomycetes | Planctomycetia | Planctomycetales | Planctomycetaceae | Gemmata |  | | * | | |  | |
| OTU_268 | Bacteria | Bacteroidetes | Sphingobacteriia | Sphingobacteriales | Sphingobacteriaceae | NA |  | |  | | | * | |
| OTU_269 | Bacteria | Bacteroidetes | Flavobacteriia | Flavobacteriales | Flavobacteriaceae | Stenothermobacter |  | |  | | | * | |
| OTU_270 | Bacteria | Proteobacteria | Alphaproteobacteria | Rhodospirillales | NA | NA | * | |  | | |  | |
| OTU_271 | Bacteria | Verrucomicrobia | Spartobacteria | NA | NA | NA | * | |  | | |  | |
| OTU_272 | Bacteria | Bacteroidetes | Sphingobacteriia | Sphingobacteriales | Sphingobacteriaceae | Mucilaginibacter | * | |  | | |  | |
| OTU_273 | Bacteria | Proteobacteria | Alphaproteobacteria | Caulobacterales | Caulobacteraceae | Caulobacter | * | |  | | |  | |
| OTU_274 | Bacteria | Proteobacteria | Alphaproteobacteria | Sphingomonadales | Sphingomonadaceae | NA | * | |  | | |  | |
| OTU_275 | Bacteria | Bacteroidetes | Bacteroidia | Bacteroidales | Bacteroidaceae | Bacteroides | * | |  | | |  | |
| OTU_276 | Bacteria | NA | NA | NA | NA | NA |  | | * | | |  | |
| OTU_277 | Bacteria | Proteobacteria | Betaproteobacteria | Burkholderiales | Comamonadaceae | Roseateles |  | | * | | |  | |
| OTU_278 | Bacteria | Proteobacteria | Gammaproteobacteria | Xanthomonadales | Xanthomonadaceae | Stenotrophomonas |  | | * | | |  | |
| OTU_279 | Bacteria | Verrucomicrobia | Subdivision3 | NA | NA | NA |  | | * | | |  | |
| OTU_280 | Bacteria | Acidobacteria | Acidobacteria_Gp1 | Gp1 | NA | NA |  | | * | | |  | |
| OTU_281 | Bacteria | Proteobacteria | Alphaproteobacteria | NA | NA | NA |  | | * | | |  | |
| OTU_282 | Bacteria | NA | NA | NA | NA | NA |  | | * | | |  | |
| OTU_283 | Bacteria | Saccharibacteria | NA | NA | NA | NA |  | | * | | |  | |
| OTU_284 | Bacteria | Armatimonadetes | Armatimonadia | Armatimonadales | Armatimonadaceae | Armatimonas/Armatimonadetes_gp1 |  | | * |  | | | |
| OTU_285 | Bacteria | Actinobacteria | Actinobacteria | Actinomycetales | Nocardioidaceae | Nocardioides |  | | * |  | | |  |
| OTU_286 | Bacteria | Bacteroidetes | Cytophagia | Cytophagales | Cytophagaceae | Siphonobacter |  | | * | | |  | |
| OTU_287 | Bacteria | Proteobacteria | Alphaproteobacteria | NA | NA | NA |  | | * | | |  | |
| OTU_288 | Bacteria | Proteobacteria | Gammaproteobacteria | Xanthomonadales | Xanthomonadaceae | Dokdonella |  | |  | | | * | |
| OTU_289 | Bacteria | Actinobacteria | Actinobacteria | Actinomycetales | Corynebacteriaceae | Corynebacterium |  | |  | | | * | |
| OTU_290 | Bacteria | Bacteroidetes | Sphingobacteriia | Sphingobacteriales | Chitinophagaceae | NA | * | |  | | |  | |
| OTU_291 | Bacteria | NA | NA | NA | NA | NA | * | |  | | |  | |
| OTU_292 | Bacteria | Proteobacteria | Alphaproteobacteria | Sphingomonadales | Sphingomonadaceae | Novosphingobium | * | |  | | |  | |
| OTU_293 | Bacteria | Proteobacteria | Deltaproteobacteria | Myxococcales | Kofleriaceae | Kofleria | * | |  | | |  | |
| OTU_294 | Bacteria | Verrucomicrobia | Subdivision3 | NA | NA | NA | * | |  | | |  | |
| OTU_295 | Bacteria | Armatimonadetes | Chthonomonadetes | Chthonomonadales | Chthonomonadaceae | Chthonomonas/Armatimonadetes_gp3 |  | * | | |  | | |
| OTU_296 | Bacteria | NA | NA | NA | NA | NA |  | | * | | |  | |
| OTU_297 | Bacteria | Planctomycetes | Planctomycetia | Planctomycetales | Planctomycetaceae | Blastopirellula |  | | * | | |  | |
| OTU_298 | Bacteria | Actinobacteria | Actinobacteria | Actinomycetales | Thermomonosporaceae | Actinoallomurus |  | | * | | |  | |
| OTU_299 | Bacteria | Planctomycetes | Planctomycetia | Planctomycetales | Planctomycetaceae | NA |  | | * | | |  | |
| OTU_300 | Bacteria | Proteobacteria | Alphaproteobacteria | Caulobacterales | Caulobacteraceae | Brevundimonas |  | | * | | |  | |
| OTU_301 | Bacteria | Proteobacteria | Alphaproteobacteria | Alphaproteobacteria_incertae_sedis | Rhizomicrobium | NA |  | | * | | |  | |
| OTU_302 | Bacteria | Planctomycetes | Planctomycetia | Planctomycetales | Planctomycetaceae | Blastopirellula |  | |  | | | * | |
| OTU_303 | Bacteria | Proteobacteria | Alphaproteobacteria | Rhodospirillales | Acetobacteraceae | Acidisoma |  | |  | | | * | |
| OTU_304 | Bacteria | Proteobacteria | Alphaproteobacteria | Caulobacterales | Caulobacteraceae | Phenylobacterium | * | |  | | |  | |
| OTU_305 | Bacteria | Proteobacteria | Alphaproteobacteria | Rhizobiales | Methylocystaceae | Hansschlegelia | * | |  | | |  | |
| OTU_306 | Bacteria | Bacteroidetes | Sphingobacteriia | Sphingobacteriales | Chitinophagaceae | NA | * | |  | | |  | |
| OTU_307 | Bacteria | Proteobacteria | Alphaproteobacteria | Rhizobiales | Rhizobiaceae | Rhizobium | * | |  | | |  | |
| OTU_308 | Bacteria | Bacteroidetes | Sphingobacteriia | Sphingobacteriales | Sphingobacteriaceae | Mucilaginibacter | * | |  | | |  | |
| OTU_309 | NA | NA | NA | NA | NA | NA | * | |  | | |  | |
| OTU_310 | Bacteria | Actinobacteria | Actinobacteria | Actinomycetales | Nocardiaceae | Rhodococcus |  | | * | | |  | |
| OTU_311 | Bacteria | Planctomycetes | Planctomycetia | Planctomycetales | Planctomycetaceae | Aquisphaera |  | | * | | |  | |
| OTU_312 | Bacteria | Proteobacteria | Alphaproteobacteria | Sphingomonadales | Sphingomonadaceae | Novosphingobium |  | | * | | |  | |
| OTU_313 | Bacteria | Proteobacteria | Gammaproteobacteria | Pseudomonadales | Moraxellaceae | Acinetobacter |  | | * | | |  | |
| OTU_314 | Bacteria | Proteobacteria | Gammaproteobacteria | NA | NA | NA |  | | * | | |  | |
| OTU_315 | Bacteria | Planctomycetes | Planctomycetia | Planctomycetales | Planctomycetaceae | Gemmata |  | | * | | |  | |
| OTU_316 | Bacteria | Verrucomicrobia | Spartobacteria | NA | NA | NA |  | | * | | |  | |
| OTU_317 | Bacteria | Planctomycetes | Planctomycetia | Planctomycetales | Planctomycetaceae | Blastopirellula |  | | * | | |  | |
| OTU_318 | Bacteria | Bacteroidetes | Sphingobacteriia | Sphingobacteriales | Chitinophagaceae | NA |  | |  | | | * | |
| OTU_319 | Bacteria | Bacteroidetes | Sphingobacteriia | Sphingobacteriales | NA | NA |  | |  | | | * | |
| OTU_320 | Bacteria | Saccharibacteria | NA | NA | NA | NA |  | |  | | | * | |
| OTU_321 | Bacteria | Verrucomicrobia | Verrucomicrobiae | Verrucomicrobiales | Verrucomicrobiaceae | Prosthecobacter | * | |  | | |  | |
| OTU_322 | Bacteria | Bacteroidetes | Cytophagia | Cytophagales | Cytophagaceae | Adhaeribacter | * | |  | | |  | |
| OTU_323 | Bacteria | Planctomycetes | Planctomycetia | Planctomycetales | Planctomycetaceae | NA | * | |  | | |  | |
| OTU_324 | Bacteria | Proteobacteria | Gammaproteobacteria | NA | NA | NA | * | |  | | |  | |
| OTU_325 | Bacteria | Proteobacteria | Alphaproteobacteria | Rhizobiales | Methylobacteriaceae | Methylobacterium | * | |  | | |  | |
| OTU_326 | Bacteria | Proteobacteria | Deltaproteobacteria | Myxococcales | NA | NA |  | | * | | |  | |
| OTU_327 | Bacteria | Acidobacteria | Acidobacteria_Gp16 | Gp16 | NA | NA |  | | * | | |  | |
| OTU_328 | Bacteria | NA | NA | NA | NA | NA |  | | * | | |  | |
| OTU_329 | Bacteria | Proteobacteria | Alphaproteobacteria | Rhodospirillales | NA | NA |  | | * | | |  | |
| OTU_330 | Bacteria | _WPS-2 | NA | NA | NA | NA |  | | * | | |  | |
| OTU_331 | Bacteria | Actinobacteria | Actinobacteria | Acidimicrobiales | Acidimicrobineae_incertae_sedis | Aciditerrimonas |  | | * | | |  | |
| OTU_332 | Bacteria | _WPS-2 | NA | NA | NA | NA |  | | * | | |  | |
| OTU_333 | Bacteria | division_WPS-2 | NA | NA | NA | NA |  | | * | | |  | |
| OTU_334 | Bacteria | Armatimonadetes | Chthonomonadetes | Chthonomonadales | Chthonomonadaceae | Chthonomonas/Armatimonadetes_gp3 |  | * | | |  | | |
| OTU_335 | Bacteria | Verrucomicrobia | Spartobacteria | NA | NA | NA |  | | * | | |  | |
| OTU_336 | Bacteria | Proteobacteria | Alphaproteobacteria | Rhizobiales | Bradyrhizobiaceae | Bosea |  | | * | | |  | |
| OTU_337 | Bacteria | Saccharibacteria | NA | NA | NA | NA |  | | * | | |  | |
| OTU_338 | Bacteria | Bacteroidetes | Sphingobacteriia | Sphingobacteriales | NA | NA |  | |  | | | * | |
| OTU_339 | Bacteria | Proteobacteria | Gammaproteobacteria | Thiotrichales | Piscirickettsiaceae | Methylophaga |  | |  | | | * | |
| OTU_340 | Bacteria | Proteobacteria | Gammaproteobacteria | Xanthomonadales | Sinobacteraceae | Alkanibacter |  | |  | | | * | |
| OTU_341 | Bacteria | Verrucomicrobia | Subdivision3 | NA | NA | NA |  | |  | | | * | |
| OTU_342 | Bacteria | Proteobacteria | Alphaproteobacteria | Caulobacterales | Caulobacteraceae | Asticcacaulis | * | |  | | |  | |
| OTU_343 | Bacteria | Acidobacteria | Acidobacteria_Gp4 | Gp4 | NA | NA | * | |  | | |  | |
| OTU_344 | Bacteria | Proteobacteria | Alphaproteobacteria | Sphingomonadales | Sphingomonadaceae | Novosphingobium | * | |  | | |  | |
| OTU_345 | Bacteria | Bacteroidetes | Bacteroidetes_incertae_sedis | Ohtaekwangia | NA | NA | * | |  | | |  | |
| OTU_346 | Bacteria | Proteobacteria | Gammaproteobacteria | Enterobacteriales | Enterobacteriaceae | Cedecea | * | |  | | |  | |
| OTU_347 | Bacteria | Acidobacteria | Acidobacteria_Gp1 | Gp1 | NA | NA |  | | * | | |  | |
| OTU_348 | Bacteria | Actinobacteria | Actinobacteria | Actinomycetales | Pseudonocardiaceae | Actinomycetospora |  | | * | | |  | |
| OTU_349 | Bacteria | division_WPS-2 | NA | NA | NA | NA |  | | * | | |  | |
| OTU_350 | Bacteria | Planctomycetes | Planctomycetia | Planctomycetales | Planctomycetaceae | NA |  | | * | | |  | |
| OTU_351 | Bacteria | Cyanobacteria | Chloroplast | Chloroplast | Streptophyta | NA |  | | * | | |  | |
| OTU_352 | Bacteria | Proteobacteria | Gammaproteobacteria | NA | NA | NA |  | | * | | |  | |
| OTU_353 | Bacteria | Actinobacteria | Actinobacteria | Solirubrobacterales | NA | NA |  | | * | | |  | |
| OTU_354 | Bacteria | Proteobacteria | Alphaproteobacteria | Rhodospirillales | Acetobacteraceae | NA |  | | * | | |  | |
| OTU_355 | Bacteria | Planctomycetes | Planctomycetia | Planctomycetales | Planctomycetaceae | Aquisphaera |  | | * | | |  | |
| OTU_356 | Bacteria | Proteobacteria | Gammaproteobacteria | Pseudomonadales | Moraxellaceae | Acinetobacter |  | | * | | |  | |
| OTU_357 | Bacteria | Acidobacteria | Acidobacteria_Gp1 | Gp1 | NA | NA |  | | * | | |  | |
| OTU_358 | Bacteria | Proteobacteria | Alphaproteobacteria | Caulobacterales | Caulobacteraceae | Phenylobacterium |  | | * | | |  | |
| OTU_359 | Bacteria | Actinobacteria | Actinobacteria | Solirubrobacterales | Conexibacteraceae | Conexibacter |  | | * | | |  | |
| OTU_360 | Bacteria | Bacteroidetes | Cytophagia | Cytophagales | Cytophagaceae | Spirosoma |  | | * | | |  | |
| OTU_361 | Bacteria | Firmicutes | Bacilli | Lactobacillales | Lactobacillaceae | Lactobacillus |  | | * | | |  | |
| OTU_362 | Bacteria | Bacteroidetes | Sphingobacteriia | Sphingobacteriales | Chitinophagaceae | NA |  | |  | | | * | |
| OTU_363 | Bacteria | Saccharibacteria | NA | NA | NA | NA |  | |  | | | * | |
| OTU_364 | Bacteria | Proteobacteria | Betaproteobacteria | Nitrosomonadales | Nitrosomonadaceae | Nitrosospira |  | |  | | | * | |
| OTU_365 | Bacteria | Bacteroidetes | Flavobacteriia | Flavobacteriales | Flavobacteriaceae | Myroides |  | |  | | | * | |
| OTU_366 | Bacteria | Proteobacteria | Alphaproteobacteria | Sphingomonadales | Sphingomonadaceae | Sphingomonas | * | |  | | |  | |
| OTU_367 | Bacteria | Planctomycetes | Planctomycetia | Planctomycetales | Planctomycetaceae | Gemmata | * | |  | | |  | |
| OTU_368 | Bacteria | Bacteroidetes | Cytophagia | Cytophagales | Cytophagaceae | Cytophaga |  | | * | | |  | |
| OTU_369 | Bacteria | Planctomycetes | Planctomycetia | Planctomycetales | Planctomycetaceae | Gemmata |  | | * | | |  | |
| OTU_370 | Bacteria | Planctomycetes | Planctomycetia | Planctomycetales | Planctomycetaceae | Singulisphaera |  | | * | | |  | |
| OTU_371 | Bacteria | Armatimonadetes | Armatimonadia | Armatimonadales | Armatimonadaceae | Armatimonas/Armatimonadetes_gp1 |  | * | | |  | | |
| OTU_372 | Bacteria | Actinobacteria | Actinobacteria | Actinomycetales | Sporichthyaceae | Sporichthya |  | | * | | |  | |
| OTU_373 | Bacteria | Planctomycetes | Planctomycetia | Planctomycetales | Planctomycetaceae | Aquisphaera |  | | * | | |  | |
| OTU_374 | Bacteria | Armatimonadetes | Chthonomonadetes | Chthonomonadales | Chthonomonadaceae | Chthonomonas/Armatimonadetes |  | * | | |  | | |
| OTU_375 | Bacteria | Proteobacteria | Gammaproteobacteria | Xanthomonadales | Sinobacteraceae | Alkanibacter |  | | * | | |  | |
| OTU_376 | Bacteria | Bacteroidetes | Sphingobacteriia | Sphingobacteriales | Chitinophagaceae | Sediminibacterium |  | | * | | |  | |
| OTU_377 | Bacteria | Bacteroidetes | Sphingobacteriia | Sphingobacteriales | Sphingobacteriaceae | Sphingobacterium |  | | * | | |  | |
| OTU_378 | Bacteria | Proteobacteria | Alphaproteobacteria | Sphingomonadales | Sphingomonadaceae | Sphingomonas |  | | * | | |  | |
| OTU_379 | Bacteria | Planctomycetes | Planctomycetia | Planctomycetales | Planctomycetaceae | NA |  | | * | | |  | |
| OTU_380 | Bacteria | Acidobacteria | Acidobacteria_Gp3 | Gp3 | NA | NA |  | | * | | |  | |
| OTU_381 | Bacteria | WPS-2 | NA | NA | NA | NA |  | | * | | |  | |
| OTU_382 | Bacteria | Proteobacteria | Gammaproteobacteria | Pseudomonadales | Moraxellaceae | Alkanindiges |  | |  | | | * | |
| OTU_383 | Bacteria | Proteobacteria | Gammaproteobacteria | Xanthomonadales | Xanthomonadaceae | Lysobacter |  | |  | | | * | |
| OTU_384 | Bacteria | Proteobacteria | Gammaproteobacteria | Gammaproteobacteria_incertae_sedis | Solimonas | NA |  | |  | | | * | |
| OTU_385 | Bacteria | Planctomycetes | Planctomycetia | Planctomycetales | Planctomycetaceae | Aquisphaera |  | |  | | | * | |
| OTU_386 | Bacteria | Acidobacteria | Acidobacteria_Gp10 | Gp10 | NA | NA | * | |  | | |  | |
| OTU_387 | Bacteria | Acidobacteria | Acidobacteria_Gp4 | Gp4 | NA | NA | * | |  | | |  | |
| OTU_388 | Bacteria | Planctomycetes | Planctomycetia | Planctomycetales | Planctomycetaceae | Pirellula | * | |  | | |  | |
| OTU_389 | Bacteria | Proteobacteria | Alphaproteobacteria | Rhizobiales | Hyphomicrobiaceae | Hyphomicrobium | * | |  | | |  | |
| OTU_390 | Bacteria | Proteobacteria | Deltaproteobacteria | Myxococcales | Polyangiaceae | Chondromyces | * | |  | | |  | |
| OTU_391 | Bacteria | Actinobacteria | Actinobacteria | Acidimicrobiales | Acidimicrobiaceae | Ilumatobacter | * | |  | | |  | |
| OTU_392 | Bacteria | Verrucomicrobia | Spartobacteria | NA | NA | NA | * | |  | | |  | |
| OTU_393 | Bacteria | NA | NA | NA | NA | NA |  | | * | | |  | |
| OTU_394 | Bacteria | Proteobacteria | Alphaproteobacteria | Rhodospirillales | Rhodospirillaceae | Dongia |  | | * | | |  | |
| OTU_395 | Bacteria | Planctomycetes | Planctomycetia | Planctomycetales | Planctomycetaceae | Aquisphaera |  | | * | | |  | |
| OTU_396 | Bacteria | Planctomycetes | Planctomycetia | Planctomycetales | Planctomycetaceae | Aquisphaera |  | | * | | |  | |
| OTU_397 | Bacteria | Acidobacteria | Acidobacteria_Gp2 | Gp2 | NA | NA |  | | * | | |  | |
| OTU_398 | Bacteria | Proteobacteria | Betaproteobacteria | Burkholderiales | NA | NA |  | | * | | |  | |
| OTU_399 | Bacteria | Planctomycetes | Planctomycetia | Planctomycetales | Planctomycetaceae | Aquisphaera |  | | * | | |  | |
| OTU_400 | Bacteria | NA | NA | NA | NA | NA |  | | * | | |  | |
| OTU_401 | Bacteria | Proteobacteria | Deltaproteobacteria | Bdellovibrionales | Bdellovibrionaceae | Bdellovibrio |  | | * | | |  | |
| OTU_402 | Bacteria | Proteobacteria | Alphaproteobacteria | Rhodospirillales | Acetobacteraceae | NA |  | | * | | |  | |
| OTU_403 | Bacteria | Proteobacteria | Alphaproteobacteria | Rhizobiales | Xanthobacteraceae | Pseudolabrys |  | |  | | | * | |
| OTU_404 | Bacteria | Proteobacteria | Alphaproteobacteria | Rhodospirillales | Acetobacteraceae | NA |  | |  | | | * | |
| OTU_405 | Bacteria | Actinobacteria | Actinobacteria | Actinomycetales | Microbacteriaceae | NA |  | |  | | | * | |
| OTU_406 | Bacteria | _WPS-2 | NA | NA | NA | NA | * | |  | | |  | |
| OTU_407 | Bacteria | Acidobacteria | Acidobacteria_Gp3 | Candidatus_Solibacter | NA | NA | * | |  | | |  | |
| OTU_408 | Bacteria | Proteobacteria | Gammaproteobacteria | Pseudomonadales | Pseudomonadaceae | Cellvibrio | * | |  | | |  | |
| OTU_409 | Bacteria | Gemmatimonadetes | Gemmatimonadetes | Gemmatimonadales | Gemmatimonadaceae | Gemmatimonas | * | |  | | |  | |
| OTU_410 | Bacteria | Planctomycetes | Planctomycetia | Planctomycetales | Planctomycetaceae | NA | * | |  | | |  | |
| OTU_411 | Bacteria | Acidobacteria | Acidobacteria_Gp1 | Gp1 | NA | NA | * | |  | | |  | |
| OTU_412 | Bacteria | Verrucomicrobia | Subdivision3 | NA | NA | NA | * | |  | | |  | |
| OTU_413 | Bacteria | Saccharibacteria | NA | NA | NA | NA |  | | * | | |  | |
| OTU_414 | Bacteria | Saccharibacteria | NA | NA | NA | NA |  | | * | | |  | |
| OTU_415 | Bacteria | Actinobacteria | Actinobacteria | Actinomycetales | Thermomonosporaceae | Actinoallomurus |  | | * | | |  | |
| OTU_416 | Bacteria | Acidobacteria | Acidobacteria_Gp1 | Gp1 | NA | NA |  | | * | | |  | |
| OTU_417 | Bacteria | Proteobacteria | Alphaproteobacteria | Caulobacterales | Caulobacteraceae | Phenylobacterium |  | | * | | |  | |
| OTU_418 | Bacteria | Proteobacteria | Deltaproteobacteria | Myxococcales | Nannocystaceae | Nannocystis |  | | * | | |  | |
| OTU_419 | Bacteria | Proteobacteria | Gammaproteobacteria | Xanthomonadales | Xanthomonadaceae | NA |  | | * | | |  | |
| OTU_420 | Bacteria | Deinococcus-Thermus | Deinococci | Thermales | Thermaceae | Thermus |  | | * | | |  | |
| OTU_421 | Bacteria | Armatimonadetes | Armatimonadia | Armatimonadales | Armatimonadaceae | Armatimonas/Armatimonadetes_gp1 |  | * | | |  | | |
| OTU_422 | Bacteria | Planctomycetes | Planctomycetia | Planctomycetales | Planctomycetaceae | NA |  | | * | | |  | |
| OTU_423 | Bacteria | Proteobacteria | Alphaproteobacteria | Rhodospirillales | Rhodospirillaceae | Magnetospirillum |  | | * | | |  | |
| OTU_424 | Bacteria | Planctomycetes | Planctomycetia | Planctomycetales | Planctomycetaceae | Aquisphaera |  | |  | | | * | |
| OTU_425 | Bacteria | Proteobacteria | Deltaproteobacteria | Bdellovibrionales | Bacteriovoracaceae | Peredibacter | * | |  | | |  | |
| OTU_426 | Bacteria | Proteobacteria | Gammaproteobacteria | Alteromonadales | Alteromonadaceae | Haliea | * | |  | | |  | |
| OTU_427 | Bacteria | Planctomycetes | Planctomycetia | Planctomycetales | Planctomycetaceae | Planctomyces | * | |  | | |  | |
| OTU_428 | Bacteria | Proteobacteria | Deltaproteobacteria | Myxococcales | Haliangiaceae | Haliangium | * | |  | | |  | |
| OTU_429 | Bacteria | Planctomycetes | Planctomycetia | Planctomycetales | Planctomycetaceae | Aquisphaera |  | | * | | |  | |
| OTU_430 | Bacteria | Proteobacteria | Gammaproteobacteria | Pasteurellales | Pasteurellaceae | Haemophilus |  | | * | | |  | |
| OTU_431 | Bacteria | Acidobacteria | Acidobacteria_Gp6 | Gp6 | NA | NA |  | | * | | |  | |
| OTU_432 | Bacteria | Proteobacteria | Deltaproteobacteria | Myxococcales | Polyangiaceae | NA |  | | * | | |  | |
| OTU_433 | Bacteria | Planctomycetes | Planctomycetia | Planctomycetales | Planctomycetaceae | Gemmata |  | | * | | |  | |
| OTU_434 | Bacteria | Firmicutes | Clostridia | Clostridiales | Clostridiales_Incertae_Sedis_XI | Anaerococcus |  | | * | | |  | |
| OTU_435 | Bacteria | Acidobacteria | Acidobacteria_Gp4 | NA | NA | NA |  | | * | | |  | |
| OTU_436 | Bacteria | Proteobacteria | Betaproteobacteria | Neisseriales | Neisseriaceae | Neisseria |  | | * | | |  | |
| OTU_437 | Bacteria | Bacteroidetes | Flavobacteriia | Flavobacteriales | Flavobacteriaceae | Chryseobacterium |  | | * | | |  | |
| OTU_438 | Bacteria | Bacteroidetes | Flavobacteriia | Flavobacteriales | Cryomorphaceae | Fluviicola |  | |  | | | * | |
| OTU_439 | Bacteria | NA | NA | NA | NA | NA | * | |  | | |  | |
| OTU_440 | Bacteria | Bacteroidetes | Bacteroidia | Bacteroidales | Bacteroidaceae | Bacteroides | * | |  | | |  | |
| OTU_441 | Bacteria | Bacteroidetes | Flavobacteriia | Flavobacteriales | Flavobacteriaceae | Elizabethkingia | * | |  | | |  | |
| OTU_442 | Bacteria | Proteobacteria | Alphaproteobacteria | Sphingomonadales | Sphingomonadaceae | Sphingomonas | * | |  | | |  | |
| OTU_443 | Bacteria | Actinobacteria | Actinobacteria | Actinomycetales | Corynebacteriaceae | Corynebacterium | * | |  | | |  | |
| OTU_444 | Bacteria | Bacteroidetes | Sphingobacteriia | Sphingobacteriales | Sphingobacteriaceae | Sphingobacterium |  | | * | | |  | |
| OTU_445 | Bacteria | NA | NA | NA | NA | NA |  | | * | | |  | |
| OTU_446 | Bacteria | Saccharibacteria | NA | NA | NA | NA |  | | * | | |  | |
| OTU_447 | Bacteria | Proteobacteria | Gammaproteobacteria | NA | NA | NA |  | | * | | |  | |
| OTU_448 | Bacteria | Planctomycetes | Planctomycetia | Planctomycetales | Planctomycetaceae | Zavarzinella |  | | * | | |  | |
| OTU_449 | Bacteria | Proteobacteria | Deltaproteobacteria | NA | NA | NA |  | | * | | |  | |
| OTU_450 | Bacteria | Firmicutes | Bacilli | Lactobacillales | Lactobacillaceae | Lactobacillus |  | | * | | |  | |
| OTU_451 | Bacteria | Verrucomicrobia | Opitutae | Opitutales | Opitutaceae | Opitutus |  | | * | | |  | |
| OTU_452 | Bacteria | Proteobacteria | Alphaproteobacteria | Rhizobiales | NA | NA |  | | * | | |  | |
| OTU_453 | Bacteria | Firmicutes | Bacilli | Lactobacillales | Streptococcaceae | Streptococcus |  | | * | | |  | |
| OTU_454 | Bacteria | Proteobacteria | Gammaproteobacteria | Xanthomonadales | Xanthomonadaceae | Dyella |  | |  | | | * | |
| OTU_455 | Bacteria | Proteobacteria | Alphaproteobacteria | Rhizobiales | Hyphomicrobiaceae | Pelagibacterium |  | |  | | | * | |
| OTU_456 | Bacteria | Planctomycetes | Planctomycetia | Planctomycetales | Planctomycetaceae | NA |  | |  | | | * | |
| OTU_457 | Bacteria | Firmicutes | Bacilli | Bacillales | Planococcaceae | Lysinibacillus |  | |  | | | * | |
| OTU_458 | Bacteria | Proteobacteria | Alphaproteobacteria | Sphingomonadales | Erythrobacteraceae | Altererythrobacter | * | |  | | |  | |
| OTU_459 | Bacteria | Proteobacteria | Alphaproteobacteria | NA | NA | NA | * | |  | | |  | |
| OTU_460 | Bacteria | Proteobacteria | NA | NA | NA | NA | * | |  | | |  | |
| OTU_461 | Bacteria | Proteobacteria | Betaproteobacteria | NA | NA | NA | * | |  | | |  | |
| OTU_462 | Bacteria | Proteobacteria | NA | NA | NA | NA | * | |  | | |  | |
| OTU_463 | NA | NA | NA | NA | NA | NA | * | |  | | |  | |
| OTU_464 | Archaea | NA | NA | NA | NA | NA | * | |  | | |  | |
| OTU_465 | Bacteria | Bacteroidetes | Bacteroidia | Bacteroidales | Bacteroidaceae | Bacteroides | * | |  | | |  | |
| OTU_466 | Bacteria | NA | NA | NA | NA | NA | * | | * | | |  | |
| OTU_467 | Bacteria | Firmicutes | Clostridia | Clostridiales | Clostridiales_Incertae_Sedis_XI | Peptoniphilus | * | | * | | |  | |
| OTU_468 | Bacteria | Acidobacteria | Acidobacteria_Gp5 | Gp5 | NA | NA |  | | * | | |  | |
| OTU_469 | Bacteria | Bacteroidetes | Bacteroidetes_incertae_sedis | Ohtaekwangia | NA | NA |  | | * | | |  | |
| OTU_470 | NA | NA | NA | NA | NA | NA |  | | * | | |  | |
| OTU_471 | Bacteria | Planctomycetes | Planctomycetia | Planctomycetales | Planctomycetaceae | Planctomyces |  | | * | | |  | |
| OTU_472 | Bacteria | Proteobacteria | Gammaproteobacteria | NA | NA | NA |  | | * | | |  | |
| OTU_473 | Bacteria | Bacteroidetes | Flavobacteriia | Flavobacteriales | Flavobacteriaceae | Flavobacterium |  | | * | | |  | |
| OTU_474 | Bacteria | Firmicutes | Clostridia | Clostridiales | Clostridiales_Incertae_Sedis_XI | Finegoldia |  | | * | | |  | |
| OTU_475 | Bacteria | Planctomycetes | Planctomycetia | Planctomycetales | Planctomycetaceae | Gemmata |  | | * | | |  | |
| OTU_476 | Bacteria | Proteobacteria | Deltaproteobacteria | NA | NA | NA |  | |  | | | * | |
| OTU_477 | Bacteria | Saccharibacteria | NA | NA | NA | NA |  | |  | | | * | |
| OTU_478 | Bacteria | Cyanobacteria | Chloroplast | Chloroplast | Streptophyta | NA |  | |  | | | * | |
| OTU_479 | Bacteria | Bacteroidetes | Sphingobacteriia | Sphingobacteriales | Chitinophagaceae | NA |  | |  | | | * | |
| OTU_480 | Bacteria | Planctomycetes | Planctomycetia | Planctomycetales | Planctomycetaceae | NA | * | |  | | |  | |
| OTU_481 | Bacteria | Armatimonadetes | Armatimonadetes_gp4 | NA | NA | NA | * | |  | | |  | |
| OTU_482 | Bacteria | Ignavibacteriae | Ignavibacteria | Ignavibacteriales | Ignavibacteriaceae | Ignavibacterium | * | |  | | |  | |
| OTU_483 | Bacteria | Proteobacteria | Deltaproteobacteria | Myxococcales | Polyangiaceae | Jahnella | * | |  | | |  | |
| OTU_484 | Bacteria | Bacteroidetes | Sphingobacteriia | Sphingobacteriales | Chitinophagaceae | NA | * | |  | | |  | |
| OTU_485 | Bacteria | Bacteroidetes | Flavobacteriia | Flavobacteriales | Flavobacteriaceae | Flavobacterium | * | |  | | |  | |
| OTU_486 | Bacteria | Proteobacteria | Gammaproteobacteria | Pseudomonadales | Pseudomonadaceae | Pseudomonas | * | |  | | |  | |
| OTU_487 | Bacteria | Proteobacteria | Alphaproteobacteria | Rhizobiales | Hyphomicrobiaceae | Devosia | * | |  | | |  | |
| OTU_488 | Bacteria | Synergistetes | Synergistia | Synergistales | Synergistaceae | Cloacibacillus | * | |  | | |  | |
| OTU_489 | Bacteria | Gemmatimonadetes | Gemmatimonadetes | Gemmatimonadales | Gemmatimonadaceae | Gemmatimonas | * | |  | | |  | |
| OTU_490 | Bacteria | Proteobacteria | Alphaproteobacteria | Rhizobiales | NA | NA | * | |  | | |  | |
| OTU_491 | Bacteria | Actinobacteria | Actinobacteria | Actinomycetales | Dermabacteraceae | Brachybacterium |  | | * | | |  | |
| OTU_492 | Bacteria | Acidobacteria | Acidobacteria_Gp1 | Gp1 | NA | NA |  | | * | | |  | |
| OTU_493 | Bacteria | Actinobacteria | Actinobacteria | Acidimicrobiales | Acidimicrobineae_incertae_sedis | Aciditerrimonas |  | | * | | |  | |
| OTU_494 | Bacteria | Firmicutes | Negativicutes | Selenomonadales | NA | NA |  | | * | | |  | |
| OTU_495 | Bacteria | Proteobacteria | NA | NA | NA | NA |  | | * | | |  | |
| OTU_496 | Bacteria | Bacteroidetes | Bacteroidia | Bacteroidales | Porphyromonadaceae | Parabacteroides |  | | * | | |  | |
| OTU_497 | Bacteria | Proteobacteria | Alphaproteobacteria | Rhizobiales | NA | NA |  | | * | | |  | |
| OTU_498 | Bacteria | NA | NA | NA | NA | NA |  | | * | | |  | |
| OTU_499 | Bacteria | Verrucomicrobia | Opitutae | Opitutales | Opitutaceae | Opitutus |  | | * | | |  | |
| OTU_500 | Bacteria | NA | NA | NA | NA | NA |  | |  | | | * | |
| OTU_501 | Bacteria | Bacteroidetes | Bacteroidia | Bacteroidales | Bacteroidaceae | Bacteroides |  | |  | | | * | |
| OTU_502 | Bacteria | Chloroflexi | Thermomicrobia | Sphaerobacterales | Sphaerobacteraceae | Sphaerobacter |  | |  | | | * | |
| OTU_503 | Bacteria | NA | NA | NA | NA | NA |  | |  | | | * | |
| OTU_504 | Bacteria | Nitrospirae | Nitrospira | Nitrospirales | Nitrospiraceae | Nitrospira |  | |  | | | * | |
| OTU_505 | Bacteria | Cyanobacteria | Chloroplast | Chloroplast | Chlorophyta | NA |  | |  | | | * | |
| OTU_506 | Bacteria | Proteobacteria | Betaproteobacteria | NA | NA | NA |  | |  | | | * | |
| OTU_507 | Bacteria | Firmicutes | Clostridia | Clostridiales | Clostridiales_Incertae_Sedis_XI | Peptoniphilus | * | |  | | |  | |
| OTU_508 | Bacteria | Verrucomicrobia | Verrucomicrobiae | Verrucomicrobiales | Verrucomicrobiaceae | Prosthecobacter | * | |  | | |  | |
| OTU_509 | Bacteria | NA | NA | NA | NA | NA | * | |  | | |  | |
| OTU_510 | Bacteria | Bacteroidetes | Flavobacteriia | Flavobacteriales | Flavobacteriaceae | Flavobacterium | * | |  | | |  | |
| OTU_511 | Bacteria | Saccharibacteria | NA | NA | NA | NA | * | |  | | |  | |
| OTU_512 | Bacteria | Cyanobacteria | NA | NA | NA | NA | * | |  | | |  | |
| OTU_513 | Bacteria | Saccharibacteria | NA | NA | NA | NA | * | |  | | |  | |
| OTU_514 | Bacteria | division_WPS-1 | NA | NA | NA | NA | * | |  | | |  | |
| OTU_515 | Bacteria | Verrucomicrobia | Opitutae | Opitutales | Opitutaceae | Opitutus | * | |  | | |  | |
| OTU_516 | NA | NA | NA | NA | NA | NA | * | |  | | |  | |
| OTU_517 | Bacteria | Bacteroidetes | Cytophagia | Cytophagales | Cytophagaceae | Cytophaga | * | |  | | |  | |
| OTU_518 | Bacteria | NA | NA | NA | NA | NA | * | |  | | |  | |
| OTU_519 | Bacteria | Firmicutes | Clostridia | Clostridiales | Lachnospiraceae | NA |  | | * | | |  | |
| OTU_520 | Bacteria | Proteobacteria | NA | NA | NA | NA |  | | * | | |  | |
| OTU_521 | Archaea | Thaumarchaeota | Nitrososphaerales | Nitrososphaeraceae | Nitrososphaera | NA |  | | * | | |  | |
| OTU_522 | Bacteria | Proteobacteria | Gammaproteobacteria | Legionellales | Coxiellaceae | Aquicella |  | | * | | |  | |
| OTU_523 | Bacteria | Verrucomicrobia | Verrucomicrobiae | Verrucomicrobiales | Verrucomicrobiaceae | Roseimicrobium |  | | * | | |  | |
| OTU_524 | NA | NA | NA | NA | NA | NA |  | | * | | |  | |
| OTU_525 | Bacteria | Acidobacteria | Acidobacteria_Gp1 | Gp1 | NA | NA |  | | * | | |  | |
| OTU_526 | Bacteria | NA | NA | NA | NA | NA |  | |  | | | * | |
| OTU_527 | Bacteria | Proteobacteria | Deltaproteobacteria | Myxococcales | Polyangiaceae | NA |  | |  | | | * | |
| OTU_528 | Bacteria | Bacteroidetes | Sphingobacteriia | Sphingobacteriales | Sphingobacteriaceae | Pedobacter |  | |  | | | * | |
| OTU_529 | Bacteria | Deinococcus-Thermus | Deinococci | Deinococcales | Deinococcaceae | Deinococcus |  | |  | | | * | |
| OTU_530 | Bacteria | Proteobacteria | Betaproteobacteria | NA | NA | NA |  | |  | | | * | |
| OTU_531 | Bacteria | NA | NA | NA | NA | NA |  | |  | | | * | |
| OTU_532 | Bacteria | Planctomycetes | Planctomycetia | Planctomycetales | Planctomycetaceae | Schlesneria | * | |  | | |  | |
| OTU_533 | Bacteria | NA | NA | NA | NA | NA | * | |  | | |  | |
| OTU_534 | Bacteria | Proteobacteria | NA | NA | NA | NA | * | |  | | |  | |
| OTU_535 | Bacteria | Proteobacteria | Gammaproteobacteria | Chromatiales | Chromatiaceae | NA | * | |  | | |  | |
| OTU_536 | Archaea | Thaumarchaeota | Nitrososphaerales | Nitrososphaeraceae | Nitrososphaera | NA | * | |  | | |  | |
| OTU_537 | Archaea | Thaumarchaeota | Nitrososphaerales | Nitrososphaeraceae | Nitrososphaera | NA | * | |  | | |  | |
| OTU_538 | Bacteria | Bacteroidetes | Cytophagia | Cytophagales | Cytophagaceae | Fibrella | * | |  | | |  | |
| OTU_539 | Bacteria | Proteobacteria | Gammaproteobacteria | Xanthomonadales | Sinobacteraceae | Alkanibacter | * | |  | | |  | |
| OTU_540 | Bacteria | NA | NA | NA | NA | NA | * | |  | | |  | |
| OTU_541 | Bacteria | NA | NA | NA | NA | NA | * | |  | | |  | |
| OTU_542 | Bacteria | Acidobacteria | Acidobacteria_Gp3 | Gp3 | NA | NA | * | |  | | |  | |
| OTU_543 | Bacteria | Gemmatimonadetes | Gemmatimonadetes | Gemmatimonadales | Gemmatimonadaceae | Gemmatimonas | * | |  | | |  | |
| OTU_544 | Bacteria | NA | NA | NA | NA | NA | * | |  | | |  | |
| OTU_545 | NA | NA | NA | NA | NA | NA | * | |  | | |  | |
| OTU_546 | Bacteria | NA | NA | NA | NA | NA | * | |  | | |  | |
| OTU_547 | Bacteria | Proteobacteria | Alphaproteobacteria | Sphingomonadales | Sphingomonadaceae | Novosphingobium | * | |  | | |  | |
| OTU_548 | Bacteria | NA | NA | NA | NA | NA | * | |  | | |  | |
| OTU_549 | Bacteria | Planctomycetes | Planctomycetia | Planctomycetales | Planctomycetaceae | Aquisphaera | * | |  | | |  | |
| OTU_550 | Bacteria | NA | NA | NA | NA | NA | * | |  | | |  | |
| OTU_551 | Bacteria | NA | NA | NA | NA | NA | * | |  | | |  | |
| OTU_552 | Bacteria | NA | NA | NA | NA | NA | * | |  | | |  | |
| OTU_553 | Bacteria | Proteobacteria | Gammaproteobacteria | NA | NA | NA | * | |  | | |  | |
| OTU_554 | Bacteria | Acidobacteria | Acidobacteria | Terriglobus | NA | NA | * | |  | | |  | |
| OTU_555 | Bacteria | Proteobacteria | Alphaproteobacteria | NA | NA | NA |  | | * | | |  | |
| OTU_556 | Bacteria | Planctomycetes | Planctomycetia | Planctomycetales | Planctomycetaceae | NA |  | | * | | |  | |
| OTU_557 | Bacteria | Acidobacteria | Acidobacteria | Gp1 | NA | NA |  | | * | | |  | |
| OTU_558 | Bacteria | Bacteroidetes | Cytophagia | Cytophagales | Flammeovirgaceae | NA |  | | * | | |  | |
| OTU_559 | Bacteria | Planctomycetes | Planctomycetia | Planctomycetales | Planctomycetaceae | Pirellula |  | |  | | | * | |
| OTU_560 | Bacteria | Verrucomicrobia | Verrucomicrobiae | Verrucomicrobiales | Verrucomicrobiaceae | NA |  | |  | | | * | |
| OTU_561 | Archaea | NA | NA | NA | NA | NA | * | |  | | |  | |
| OTU_562 | Bacteria | NA | NA | NA | NA | NA | * | |  | | |  | |
| OTU_563 | Bacteria | NA | NA | NA | NA | NA | * | |  | | |  | |
| OTU_564 | NA | NA | NA | NA | NA | NA | * | |  | | |  | |
| OTU_565 | Bacteria | Proteobacteria | Alphaproteobacteria | Caulobacterales | Caulobacteraceae | Asticcacaulis | * | |  | | |  | |
| OTU_566 | Bacteria | Proteobacteria | Gammaproteobacteria | Xanthomonadales | Xanthomonadaceae | Stenotrophomonas | * | |  | | |  | |
| OTU_567 | Bacteria | Proteobacteria | Betaproteobacteria | Burkholderiales | Oxalobacteraceae | Massilia | * | |  | | |  | |
| OTU_568 | Bacteria | Proteobacteria | Alphaproteobacteria | Caulobacterales | Caulobacteraceae | Asticcacaulis | * | |  | | |  | |
| OTU_569 | Bacteria | Planctomycetes | Planctomycetia | Planctomycetales | Planctomycetaceae | NA | * | |  | | |  | |
| OTU_570 | Bacteria | Proteobacteria | Alphaproteobacteria | NA | NA | NA | * | |  | | |  | |
| OTU_571 | Bacteria | Actinobacteria | Actinobacteria | Acidimicrobiales | Iamiaceae | Iamia | * | |  | | |  | |
| OTU_572 | Bacteria | Proteobacteria | NA | NA | NA | NA | * | |  | | |  | |
| OTU_573 | Bacteria | Actinobacteria | Actinobacteria | Bifidobacteriales | Bifidobacteriaceae | Bifidobacterium | * | |  | | |  | |
| OTU_574 | Bacteria | NA | NA | NA | NA | NA | * | |  | | |  | |
| OTU_575 | Bacteria | NA | NA | NA | NA | NA | * | |  | | |  | |
| OTU_576 | Bacteria | NA | NA | NA | NA | NA | * | |  | | |  | |
